# Rational Design of Carbon Aerogels for Alkali-Metal-Ion Batteries: Controlled Synthesis, Heteroatom Doping, and Energy Storage Applications

**DOI:** 10.3390/gels12060553

**Published:** 2026-06-19

**Authors:** Anrui Li, Simin Hua, Le Sun, Qinsi Shao, Delun Zhu, Ruicheng Bai

**Affiliations:** 1 Research Center for Composite Materials, School of Materials Science and Engineering, Shanghai University, Shanghai 200072, China; 18663621362@shu.edu.cn (A.L.); huasm@shu.edu.cn (S.H.); 2 State Key Laboratory of Advanced Refractories, Shanghai University, Shanghai 200444, China; 3 Shaoxing Institute of Technology, Shanghai University, Shaoxing 312000, China; sunle321@126.com; 4 Institute for Sustainable Energy, School of Sciences, Shanghai University, Shanghai 200444, China; qinsishao@shu.edu.cn; 5 Department of Polymer Science and Engineering, Zhejiang University, Hangzhou 310058, China

**Keywords:** carbon aerogels, controlled synthesis, heteroatom doping, alkali-metal-ion batteries

## Abstract

Carbon aerogels possess continuous three-dimensional conductive networks, hierarchical pore architectures, and tunable surface chemistry. These structural characteristics make them suitable electrode materials for alkali-metal-ion batteries. This review examines the controlled synthesis and heteroatom doping of carbon aerogels. The discussion links framework construction, electronic-structure modulation, and storage mechanism matching with their electrochemical behavior. The rational design of carbon aerogels should move beyond the simple pursuit of high specific surface area or high dopant content. Effective electrodes require the coordinated regulation of pore architecture, conductive continuity, heteroatom-doped sites, and ion-storage pathways. The current application status of carbon aerogels in alkali-metal-ion batteries is also analyzed from an industrial perspective. A mechanism-oriented and application-oriented framework is therefore required to translate carbon aerogel-based electrodes from structural optimization to a practical battery.

## 1. Introduction

Global energy consumption continues to rise, and environmental sustainability places stricter requirements on electrochemical energy storage devices. Advanced devices are expected to deliver high energy density, high power density, long cycling life, safety, and operational stability [1,2,3]. However, many electrode materials still suffer from sluggish ion transport, discontinuous electron conduction, unstable electrode/electrolyte interfaces, and structural degradation during repeated cycling. These problems make it difficult to achieve high capacity, fast charge/discharge capability, and long-term durability at the same time [4,5,6]. The electrochemical performance of electrode materials is strongly related to pore architecture, conductive continuity, surface chemistry, and structural evolution during cycling. Effective electrode materials should integrate open ion transport channels, continuous conductive networks, robust porous frameworks, and tunable interfacial chemistry. This structural and chemical integration is essential for improving electrochemical energy storage performance [7,8].

Carbon aerogels are three-dimensional porous carbon materials constructed from interconnected nanoscale carbon frameworks. They are characterized by continuous conductive networks, hierarchical pore architectures, low density, and tailorable surface chemistry [9,10,11]. Carbon aerogels have been widely investigated as functional electrode materials for electrochemical energy storage and related fields because of these structural and chemical features [12,13]. Their pore architecture and surface chemistry can be regulated through sol–gel chemistry, drying, carbonization, activation, and heteroatom doping. This tunability allows carbon aerogels to adapt to different charge-storage mechanisms [14,15]. Catalyst selection directly affects polymerization kinetics, crosslinking density, and primary particle size during sol–gelation. The pore-size distribution and framework connectivity of the resulting aerogels are governed by these parameters [16,17]. Drying routes, including supercritical drying, freeze-drying, and ambient-pressure drying, usually require a balance among pore-structure preservation, process complexity, and preparation cost [18,19]. Physical or chemical activation can promote the development of micro/mesoporous structures, increase accessible surface area, and optimize ion transport pathways [20,21]. In addition to pore regulation, single heteroatom doping or multi-heteroatom doping with elements such as nitrogen, phosphorus, sulfur, and boron can modulate the local electronic structure of carbon frameworks. Additional active sites can be introduced, and interfacial reaction kinetics can be improved through these doping strategies. Multi-heteroatom doping may overcome the limitations of single heteroatom doping through synergistic interactions among different dopant atoms. Adsorption behavior, charge transfer, and electrochemical reversibility can be regulated more effectively.

**Figure 1:**
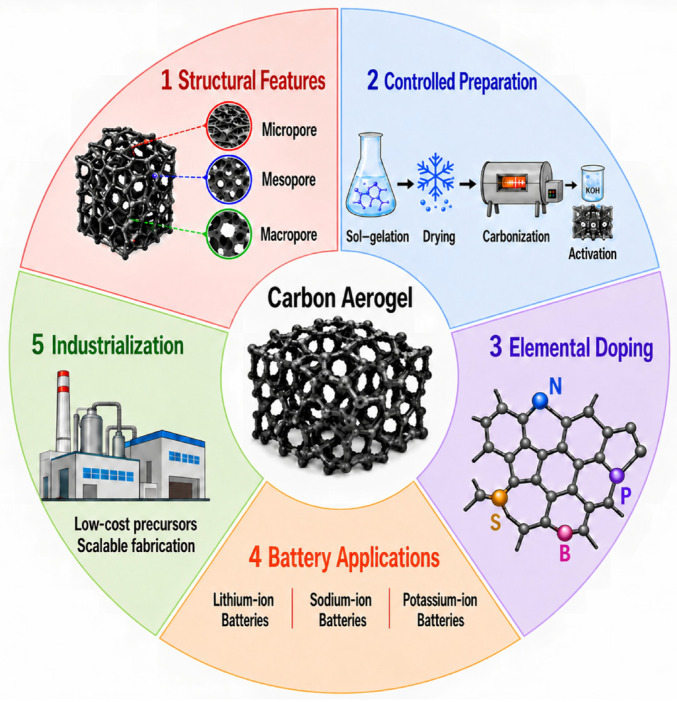
Schematic illustration for carbon aerogels, including controlled synthesis, heteroatom doping, applications in alkali-metal-ion batteries, and industrialization.

Recent studies have advanced the controlled synthesis, heteroatom doping, and energy storage applications of carbon aerogels. Existing reviews have mainly discussed carbon aerogels from the perspectives of precursor selection, preparation methods, modification strategies, specific device systems, or a relatively broad scope. To further clarify the position of the present review, representative recent reviews on carbon aerogels are compared in Table 1. However, a unified basis for comparing carbon aerogel electrodes across different alkali-metal-ion batteries remains insufficient. To clarify the distinct contribution of this review, the discussion is organized around several comparable evaluation criteria, including synthesis–structure correlation, pore accessibility, conductive-network continuity, doping configurations, ion-specific mechanisms, and scalable production. Based on these criteria, this review not only summarizes the reported structural and chemical modification strategies, but also examines whether these strategies effectively improve ion transport, charge transfer, interfacial stability, electrode-level performance, and manufacturing feasibility. High specific surface area or dopant content alone does not determine the electrochemical performance of carbon aerogels in alkali-metal-ion batteries. Electrochemical performance depends on the coordinated matching of pore architecture, conductive networks, doped elemental sites, and specific charge-storage pathways. The key issue is whether these structural and chemical features can be effectively matched with Li^+^/Na^+^/K^+^-related mechanisms under realistic electrode conditions. As illustrated in Figure 1, this review systematically discusses the rational design of carbon aerogels from the perspectives of controlled synthesis, heteroatom doping, alkali-metal-ion battery applications, and scalable development. Structural regulation strategies during sol–gelation, drying, carbonization, and activation are first summarized. The effects of single heteroatom doping and multi-heteroatom doping on electronic-structure modulation, active-site construction, and interfacial reaction behavior are then discussed. Applications in lithium-, sodium-, and potassium-ion batteries are further reviewed, with emphasis on the matching between carbon aerogel structures and different ion-storage requirements. Reported enterprise routes and scalable manufacturing considerations are finally analyzed to clarify current industrialization efforts. Major challenges in structural design, performance evaluation, and practical application are also summarized. This discussion provides guidance for the rational design and realistic use of carbon aerogel-based electrode materials in advanced energy storage systems.

## 2. Controlled Synthesis and Pore Regulation

The energy storage performance of carbon aerogels is governed by pore hierarchy and pore connectivity. Micropores mainly provide ion adsorption sites and charge-storage interfaces, while mesopores facilitate electrolyte infiltration and ion diffusion. Macropores and interconnected channels serve as rapid mass-transport pathways, especially in thick electrodes or under high-current-density conditions. Therefore, pore-structure design should not simply pursue high specific surface area. It should balance accessible active interfaces, ion/electron-transport pathways, and framework stability. In general, sol–gel assembly determines the initial network morphology, while drying controls pore-structure retention. Carbonization regulates framework shrinkage, micropore development, and local carbon ordering. Activation further reconstructs the accessible surface area and pore-size distribution [28]. Accordingly, this section discusses controllable synthesis as a sequence of network formation, pore retention, carbon framework fixation, and post-carbonization pore reconstruction.

### 2.1. Sol–Gelation

The primary network structure of carbon aerogels is mainly formed during sol–gelation. In a typical resorcinol–formaldehyde (RF) system, resorcinol and formaldehyde undergo hydroxymethylation and polycondensation under catalytic conditions. A three-dimensional crosslinked polymer network is then gradually constructed. Catalytic conditions regulate polymerization kinetics, nucleation density, cluster growth, and intercluster connection. These factors determine the primary particle size, framework connectivity, and pore-structure evolution of the resulting aerogels [29]. Therefore, sol–gelation is a key stage for controlling pore morphology, pore-size distribution, and framework robustness in carbon aerogels [27].

**Figure 2:**
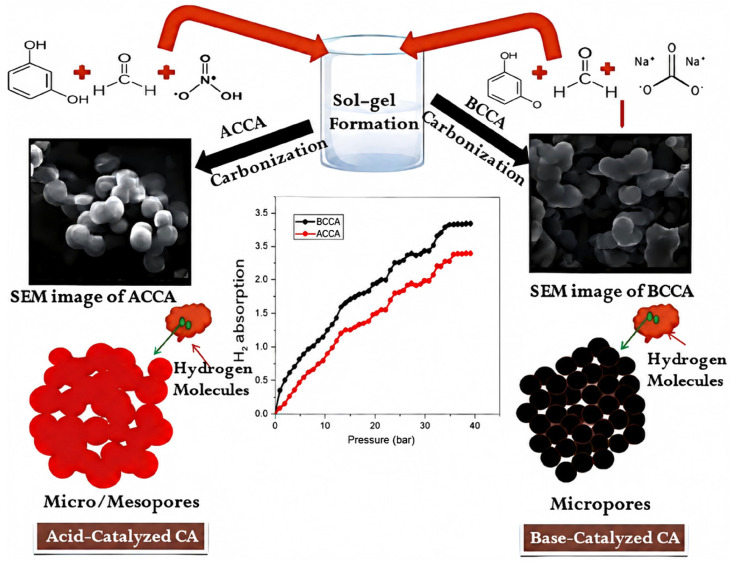
Schematic for the synthesis and hydrogen mechanism on acid-catalyzed carbon aerogel (ACCA) and base-catalyzed carbon aerogel (BCCA) formation [31].

The catalyst type directly governs the formation pathway of the RF gel network and the resulting pore morphology. Pekala first used sodium carbonate as an alkaline catalyst, which laid the foundation for RF-derived carbon aerogels [30]. Resorcinol undergoes deprotonation and then reacts with formaldehyde through hydroxymethylation and polycondensation in alkaline systems. This reaction pathway favors the formation of highly crosslinked networks assembled from interconnected particles. Pandey et al. [31] compared RF systems catalyzed by nitric acid and sodium carbonate. They found that micropores dominated the sample prepared under basic catalysis. This sample showed an average pore size of approximately 1.71 nm and a specific surface area of 461.6 m^2^ g^−1^. The sample prepared under acidic catalysis showed a hybrid pore structure containing both micropores and mesopores. Its average pore size increased to 1.90 nm, while its specific surface area decreased to 395.9 m^2^ g^−1^ (Figure 2). This difference is mainly related to the stronger protonation of hydroxymethyl derivatives and dehydration condensation under acidic conditions. These reactions increase the proportion of linear chain segments and reduce the crosslinking density of the network. The results indicate that catalyst type governs the network formation mode, pore-size distribution, and final pore morphology of RF-derived carbon aerogels.

**Figure 3:**
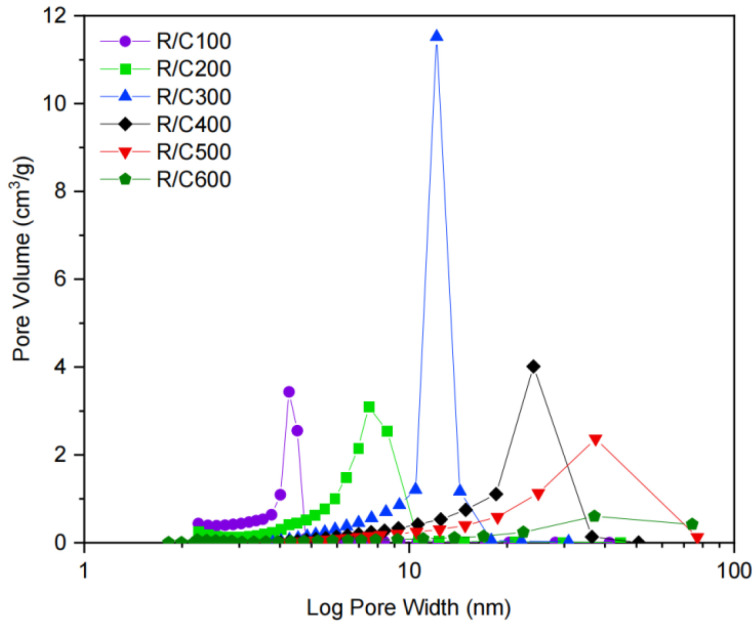
Pore-size distributions for sodium carbonate gels at resorcinol/catalyst molar ratios (R/C ratios) of 100–600 [32].

After the catalyst type has been selected, the catalyst dosage further regulates the nucleation density of the RF gel network and the characteristic length scale of the resulting pore structure. An increase in the resorcinol-to-catalyst molar ratio (R/C) reduces the catalyst concentration. Fewer active nucleation sites are then generated in the system. Under such conditions, polymeric clusters have more space to grow, and larger pores are formed. By contrast, a lower R/C ratio corresponds to a higher catalyst concentration. It leads to denser nucleation, faster monomer consumption, smaller primary particles, and a more refined pore structure. Martin et al. [32] confirmed that increasing the R/C ratio shifts the aerogel texture toward larger pores by changing nucleation density, particle packing, and framework connectivity (Figure 3).

Nevertheless, regulation based only on acid/base catalysis or the R/C ratio remains insufficient for simultaneously achieving precise pore-structure control and high framework stability. Composite catalytic systems or functional components can introduce additional variables for modulating the RF sol–gel process. Gao et al. [33] incorporated graphite crystallite nanomaterials (GCNs) into the RF system. Oxygen-containing functional groups at the edges of GCNs promoted the participation of formaldehyde in polycondensation reactions and accelerated gel formation. Meanwhile, the rigid nanosheet structure of GCNs supported the gel framework and broadened the tunable mesopore range. Theoretical calculations further showed that GCNs could lower the energy barrier of the RF addition reaction. This result indicates that nanocarbon components can regulate both reaction kinetics and network growth. Men et al. [34] introduced chitosan into an acetic acid-catalyzed system. Amino groups in chitosan reacted with formaldehyde through Schiff-base reactions, and auxiliary crosslinked structures were formed. As a result, the particle size was refined, and drying shrinkage was reduced. GCNs mainly accelerate RF addition and broaden mesopore tunability, whereas chitosan introduces auxiliary crosslinking that refines particles and suppresses drying shrinkage.

Building on these studies, the understanding of catalytic regulation has gradually shifted from conventional acid/base effects to ion-specific effects. Conventional interpretations usually attributed catalyst-induced changes in RF gel networks to pH-dependent polymerization kinetics. However, Martin et al. [32] showed that only minor changes occurred in the pore structure of the resulting materials when the Na^+^ concentration was kept constant and the pH was varied. In contrast, progressive replacement of Na^+^ with NH_4_^+^ transformed the gel network from a porous structure into a nonporous one. This observation indicates that catalyst cations not only contribute to charge balance but also participate in the regulation of cluster assembly during sol–gelation. Na^+^ can stabilize the water structure and colloidal aggregation state as a kosmotropic ion. This stabilization promotes the connection of polymeric clusters into a continuous porous network. NH_4_^+^ shows a weaker stabilizing effect on network formation, making it less favorable for porous framework construction. RF gelation is jointly governed by polymerization kinetics, cation identity, and ionic strength. Catalyst system design needs to go beyond pH control and account for the ionic environment involved in cluster assembly regulation.

For large-scale factory production, formulation design needs to be simplified, gelation time should be shortened, and process controllability must be maintained. Batch-to-batch reproducibility should also be improved during ambient-pressure drying and continuous carbonization. These improvements will support the low-cost, reproducible, and scalable production of carbon aerogels.

### 2.2. Drying

The drying process determines how effectively the wet-gel network is preserved and transferred into the final carbon aerogel during subsequent carbonization. Its central objective is to minimize capillary stress caused by solvent migration and phase transition [35]. This stress must be reduced to suppress pore shrinkage, framework deformation, and network collapse. For carbon aerogels, the commonly used drying routes mainly include supercritical drying, freeze-drying, and ambient-pressure drying. Supercritical drying eliminates the gas–liquid interface and suppresses capillary pressure. Freeze-drying reduces framework compression through a freezing–sublimation process and can reconstruct pore channels. Ambient-pressure drying lowers drying stress through solvent exchange and framework reinforcement [36]. The drying route should be selected according to the precursor network characteristics and the target pore architecture.

**Figure 4:**
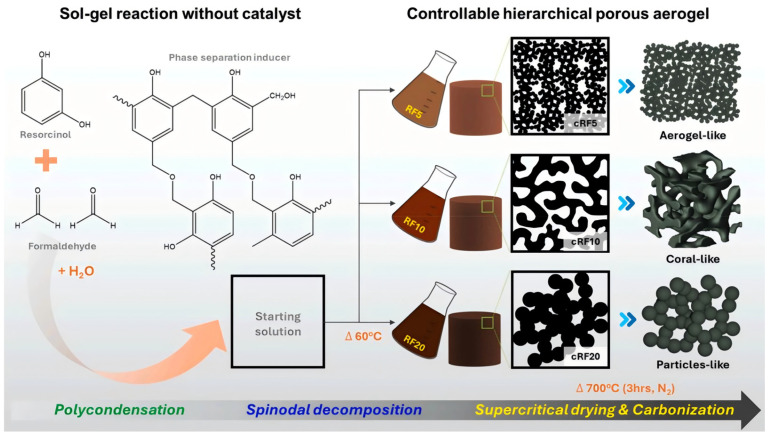
Schematic illustration of the hierarchical aerogels synthesized via the spinodal decomposition approach [39].

Supercritical drying is particularly effective in preserving the original gel network. When the drying medium reaches the supercritical state, the gas–liquid interface disappears. Interfacial-tension-induced framework compression is then markedly weakened. The three-dimensional porous structure of the wet gel can be retained with limited shrinkage [37]. Patil et al. [38] employed a high-temperature supercritical ethanol drying technique and prepared carbon aerogels in ethanol medium at 265 °C, with a linear shrinkage of only 4%, indicating that this method is suitable for the shape-preserving preparation of gels with relatively weak frameworks. Choi et al. [39] combined supercritical drying with spinodal decomposition and obtained a coral-like bicontinuous network, suggesting that this route can also effectively inherit complex hierarchical structures formed at the precursor stage (Figure 4). Overall, supercritical drying is well suited for carbon aerogel systems requiring high structural fidelity, especially fragile frameworks and complex hierarchical networks. However, its dependence on high-pressure equipment, strict process control, and high scale-up cost still limits its broader industrial application.

Compared with supercritical drying, which primarily emphasizes structural fidelity, freeze-drying relies more strongly on ice-crystal templating to reconstruct pore architectures. In this method, the solvent in the wet gel is first frozen and then removed through ice-crystal sublimation under reduced pressure, thereby avoiding liquid-phase evaporation and reducing capillary-pressure-induced framework compression [40]. During freezing, ice-crystal nucleation, growth, and solute rejection reorganize the gel network, making the resulting pore architecture jointly governed by precursor framework rigidity, freezing rate, and ice-crystal growth behavior. Thomas et al. [41] regulated the temperature gradient through an ice-templating method to induce directional ice-crystal growth, thereby constructing carbon aerogels with anisotropic channels and demonstrating the advantage of freeze-drying in oriented pore-channel formation. Kraiwattanawong et al. [42] further showed that the effectiveness of freeze-drying is closely related to the state of the precursor network. When the R/C ratio was 200, the mesopore volume of the freeze-dried sample was significantly higher than that of the evaporation-dried sample. As the R/C ratio increased to 500, the difference in pore structure between the materials obtained by the two drying methods became markedly smaller. These results indicate that freeze-drying is more suitable for constructing oriented pores, macroscopic channels, and anisotropic structures, while its pore-regulation capability depends on the coordinated matching among precursor network rigidity, freezing rate, and ice-crystal growth behavior.

The main advantages of ambient-pressure drying lie in its process feasibility and scale-up potential. This route controls drying stress within the tolerance range of the gel network by reducing solvent surface tension, reinforcing the gel framework, and optimizing the heating procedure. Therefore, its effectiveness is closely associated with precursor framework stability, the solvent-exchange pathway, and the structural reinforcement strategy. Cheng et al. [43] adopted tert-butanol (TBA) exchange combined with a gradient heating program. The low surface tension of TBA weakened capillary pressure, while the construction of a SiO_2_/RF hybrid network enhanced the mechanical strength of the gel framework, allowing the linear shrinkage to be controlled within 16.4–18.3%, significantly lower than the value above 40% commonly observed for conventional SiC/C aerogels. Kraiwattanawong et al. [42] further showed that, after TBA exchange and cotton-fiber reinforcement, the evaporation-dried samples exhibited mesopore sizes of 3.6–3.9 nm, close to those of the freeze-dried samples, with specific surface areas reaching 680–730 m^2^ g^−1^. These results show that ambient-pressure drying can approach the pore retention of freeze-drying when solvent exchange and framework reinforcement are properly matched with the precursor network.

Ambient-pressure drying is more favorable for large-scale preparation because of its low cost, simple equipment requirements, and low energy consumption. However, the resulting pore structure is strongly influenced by precursor selection, drying temperature, and drying time.

### 2.3. Carbonization

Carbonization converts the organic gel into a conductive carbon framework while changing micropore formation, framework shrinkage, defect evolution, and local carbon ordering. As the carbonization temperature increases, unstable functional groups are gradually removed, and volatile species are released. This process promotes micropore formation. At the same time, framework shrinkage and aromatic structure rearrangement occur. Further temperature increase promotes the formation of ordered carbon domains. However, excessive carbonization may cause micropore collapse or pore coalescence [44]. Different requirements for micropore development, conductive-network continuity, and graphitization degree determine the selection of carbonization temperature.

Structural evolution during carbonization involves coupled changes in micropore formation, framework shrinkage, defect evolution, and carbon ordering. A related study [38] showed that RF aerogels carbonized at 600–900 °C exhibited increased specific surface area and pore volume. Their pore structure also shifted toward micropore development. At approximately 700 °C, a pebble-like interconnected framework was observed. Carbonization reconstructed the particle connection mode and framework morphology. Yang et al. [45] investigated phenolic resin/polyurethane-urea oligomer-derived carbon aerogels. They showed that heat treatment at 800–1600 °C increased framework density, promoted closed-pore rearrangement, and induced the formation of graphitized nanocrystallites. The mechanical stability of the material was also enhanced. Thus, carbonization temperature should be selected according to the target balance among micropore development, conductive continuity, and structural retention, rather than simply increased.

### 2.4. Activation

Activation is an important post-treatment strategy that affects the pore structure and active sites of carbon aerogels. The pore-size distribution can be reconstructed by activation, and the porosity can also be increased [46]. Activation strategies are mainly divided into three categories: physical activation, chemical activation, and synergistic activation [25].

Physical activation mainly proceeds through controlled gas etching of pre-existing defects and pore walls. This method usually uses gaseous oxidants, such as CO_2_, steam, or O_2_, to selectively ablate the carbon framework at high temperature. CO_2_ is the most commonly used gaseous oxidant. Its basic reaction can be expressed as [47]

$$C+{\mathrm{CO}}_{2}\to 2\mathrm{CO}\;(△H=+173\;kJ\;{mol}^{-1})$$

**Figure 5:**
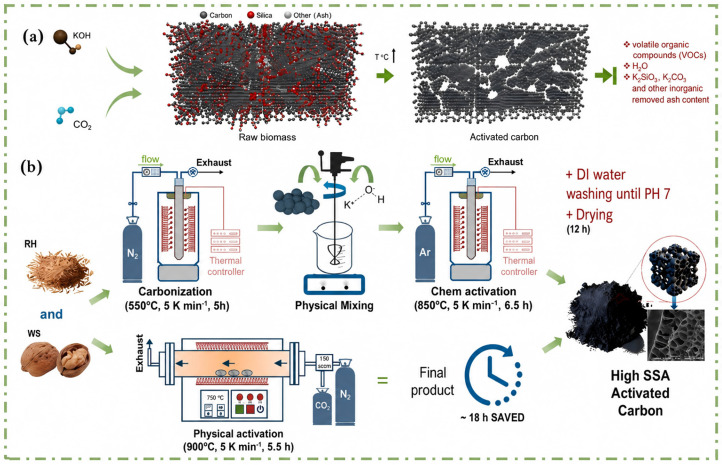
Theoretical (**a**) and practical (**b**) illustration of the preparation of activated carbon based on different biomass and activation agents (chemical vs. physical). T °C ↑ indicates an increase in temperature during the thermal activation process [48].

This reaction tends to occur preferentially at defect sites, gradually enlarging existing micropores and improving pore-channel connectivity. Taurbekov et al. [48] showed that CO_2_-activated samples exhibited a better kinetic response at high current densities, indicating that physical activation is more favorable for constructing interconnected pores and rapid mass-transport pathways, with the preparation process shown in Figure 5. Overall, physical activation involves relatively mild etching and is suitable for improving pore openness and rate performance, although its ability to generate extremely high specific surface areas remains limited when used alone.

Chemical activation creates pores more aggressively than physical activation, usually producing a higher micropore fraction after thermal treatment and washing. This method usually involves mixing carbon precursors with chemical agents such as KOH, NaOH, ZnCl_2_, or H_3_PO_4_, followed by high-temperature treatment and subsequent washing to remove residual activating agents. Taking KOH as an example, its activation process involves multiple redox and intercalation reactions. Li et al. [49] pointed out that one typical reaction pathway can be expressed as

$$4\mathrm{KOH}+C\to K_{2}{\mathrm{CO}}_{3}+K_{2}O+2H_{2}\;(△H=+186\;kJ\;{mol}^{-1}).$$

The formed K_2_O and K_2_CO_3_ can further participate in carbon etching. Metallic potassium vapor can also penetrate carbon domains and expand local interlayer spacing. As a result, micropores and mesopores are progressively developed. Related studies have shown that KOH activation can significantly enhance pore development and increase accessible charge-storage interfaces in carbon aerogels. Taurbekov et al. [48] also showed that KOH-activated samples generally possess a higher micropore fraction and larger specific surface area. However, strong chemical etching may cause excessive micropore formation, poor pore-channel connectivity, framework damage, and a heavier post-treatment burden. Ion transport under high-rate conditions can therefore be restricted. Chemical activation is more suitable for enhancing adsorption- and interface-dominated capacity contributions. In practical applications, the activating-agent dosage, washing procedure, and pore-structure regulation window should be carefully controlled.

Synergistic activation addresses the limitations of single activation routes in pore development, pore-channel connectivity, and framework stability. In this strategy, physical and chemical activation processes are coupled in a sequential or in situ manner. Murugavel et al. [47] adopted a stepwise strategy involving CO_2_ pre-activation followed by deep KOH etching. Oxygen-containing functional groups were introduced onto the carbon surface, and initial pores were generated during CO_2_ pretreatment. The resulting carbon aerogel showed a micropore fraction of 88% and a specific surface area of up to 3010 m^2^ g^−1^. This result indicates that pre-activation can improve the reaction selectivity and pore accessibility of subsequent deep chemical activation. Edathil et al. [50] proposed an in situ mineralization-assisted activation and graphitization strategy, referred to as iMAG. In this approach, KOH induced the formation of CaCO_3_ templates. CO_2_ released from CaCO_3_ decomposition enabled in situ physical activation. Residual KOH further etched the carbon framework, while CaO assisted the graphitization process and promoted the formation of a conductive porous framework. The resulting self-supporting electrode delivered a specific capacitance of 322 F g^−1^. In this way, synergistic activation combines reactive-site preconstruction, hierarchical pore generation, and partial framework stabilization within one pore-regulation route.

However, the activation degree must be controlled within an appropriate range. Excessive activation does not continuously increase effective storage sites; instead, severe etching may induce overdeveloped porosity, thinner pore walls, and even pore-structure collapse, thereby weakening pore connectivity and structural stability. Lee et al. [51] investigated the pore evolution of carbon aerogels by adjusting the CO_2_ activation time and revealed the adverse effect of excessive activation. Moderate CO_2_ activation promoted micropore formation and expanded part of the micropores into mesopores, thereby improving the surface area, pore volume, and ion accessibility of carbon aerogels. When the activation time was further extended, the pore structure was not further optimized; instead, both surface area and pore volume decreased. The authors attributed this decline to the collapse of the porous structure caused by excessive activation. Mechanistically, CO_2_ continuously reacts with the carbon framework to generate CO. Moderate etching opens pore channels and increases effective porosity, whereas prolonged activation over-consumes pore walls and causes large pores or interconnected channels to collapse. Raman results also showed that the defect density increased with activation time, but excessive defects did not further improve electrochemical performance. Instead, the overactivated carbon aerogel showed lower capacitance and rate retention, indicating that activation should balance pore generation, pore-wall stability, and ion transport.

The current difficulty is to establish a stable, low-cost, and reproducible activation process. Physical activation has a relatively simple process flow, but its activation depth is limited. Chemical activation provides efficient pore generation, but chemical pollution and residue-related issues remain concerns. Synergistic activation combines the advantages of physical and chemical activation, but it usually involves higher cost and greater operational difficulty. These issues represent the main challenges faced by industrial-scale production.

### 2.5. Summary

To provide a clearer comparison of how different synthesis and pore-regulation steps affect the structural evolution and energy storage behavior of carbon aerogels, Table 2 summarizes the main effects of sol–gelation, drying, carbonization, and activation on pore-size distribution, specific surface area, and electrochemical properties. To further compare different carbon aerogel preparation routes, Table 3 summarizes the precursor systems, gelation catalysts, drying methods, carbonization temperatures, activation strategies, and pore-structure parameters reported in representative studies. These comparisons show that pore regulation in carbon aerogels is a stepwise process. Sol–gelation defines the initial network and pore-size distribution, drying determines pore retention and shrinkage control, carbonization regulates micropore development and conductive-framework formation, and activation further reconstructs accessible pores and surface area. Therefore, carbon aerogel design should not simply pursue high specific surface area, but should balance micropore-derived storage sites, mesopore/macropore transport channels, conductive continuity, and framework stability.

## 3. Heteroatom Doping

Heteroatom doping regulates carbon aerogels by changing local charge distribution, defect chemistry, surface polarity, and interfacial reactivity. These changes further affect ion adsorption, charge transfer, wettability, and electrode/electrolyte reactions [52].

According to doping composition, heteroatom doping in carbon aerogels can generally be divided into single heteroatom doping and multi-heteroatom doping [53]. Single heteroatom doping is useful for clarifying the specific structural and electronic roles of individual dopant atoms, while multi-heteroatom doping can further regulate active-site configuration, electrical conductivity, and interfacial reaction behavior through complementary or synergistic interactions among different doped elements [54]. Therefore, the effectiveness of heteroatom doping depends not only on dopant content, but also on dopant type, bonding configuration, spatial distribution, and coupling with the pore architecture. This section discusses how single heteroatom and multi-heteroatom doping strategies regulate the energy storage performance of carbon aerogels from the perspectives of electronic-structure modulation, active-site construction, and interfacial kinetics. It should be noted that dopant atoms in carbon aerogels exhibit certain cross-system commonalities in regulating electronic structure, surface polarity, wettability, ion adsorption, and interfacial reaction activity. Therefore, when discussing doping mechanisms, this section also introduces relevant studies from supercapacitors, adsorption systems, and capacitive deionization to clarify the general regulatory roles of dopant atoms. However, for evaluating lithium-, sodium-, and potassium-ion battery performance, this review still uses experimental results obtained from the corresponding battery systems as the primary basis.

### 3.1. Single Heteroatom Doping

Single-element doping allows the contribution of each dopant to be examined separately before more complex co-doping systems are considered. In carbon aerogels, N, P, B, and S represent four typical regulation modes: electronic modulation, diffusion-environment adjustment, polarity regulation, and interfacial anchoring [53].

Nitrogen doping mainly improves energy storage reaction kinetics by regulating the local electronic structure, surface polarity, defect density, and interfacial active sites of carbon aerogels. Because the atomic radius of nitrogen is close to that of carbon, its incorporation generally does not cause severe distortion of the carbon framework, while its higher electronegativity can induce local charge redistribution and improve the wettability and electron-transport capability of the carbon surface [55]. Liao et al. [56] constructed a Co_3_O_4_@NCA (N-doped carbon aerogel) micro/nano hierarchical composite anode, in which the N-doped carbon aerogel served simultaneously as a conductive matrix and a surface coating layer. The three-dimensional aerogel network provided continuous electron-transport pathways and porous ion-diffusion channels for Co_3_O_4_, while the defects and active sites introduced by the N-doped carbon framework further enhanced interfacial reaction activity and pseudocapacitive contribution. The resulting Co_3_O_4_@NCA-1 retained a reversible capacity of 600.2 mAh g^−1^ after 200 cycles at 100 mA g^−1^, markedly outperforming pristine Co_3_O_4_. This study indicates that N-doped carbon aerogels play a role beyond conductive support in composite anodes for lithium-ion batteries. Li^+^ diffusion, electron transport, and conversion-reaction reversibility are synergistically enhanced by porous frameworks, defect sites, and interfacial coating.

**Figure 6:**
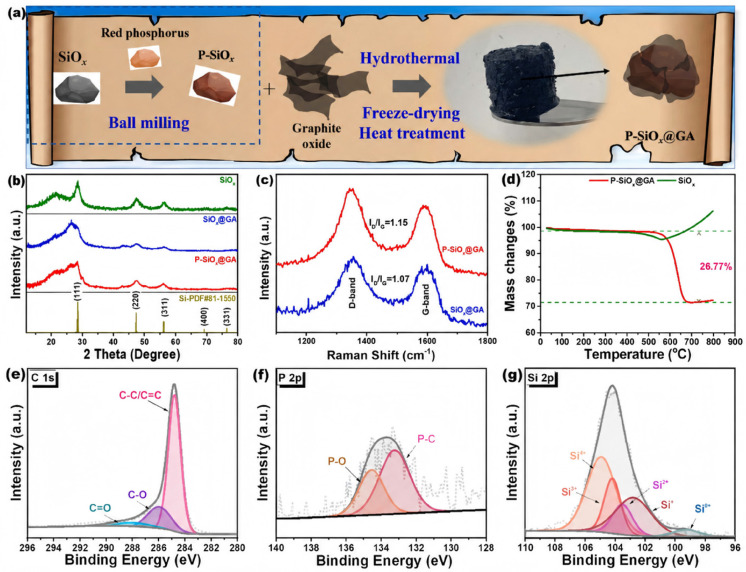
(**a**) Schematic view of the preparation of the P-SiO_x_@GA composite anode. (**b**) XRD patterns of pristine SiO_x_, SiO_x_@GA and P-SiO_x_@GA with the corresponding Si standard pattern-PDF# 81–1550. (**c**) Raman spectra of SiO_x_@GA and P-SiO_x_@GA. (**d**) TGA curves of SiO_x_ and P-SiO_x_@GA from 25 to 800 °C at a heating rate of 5 °C min^−1^ in air and XPS spectra of (**e**) Si 2p, (**f**) C 1s, and (**g**) P 2p of the P-SiO_x_@GA composite [58].

Phosphorus doping uses its larger atomic radius and P–C/P–O bonding to introduce lattice distortion, defect sites, and interfacial transport pathways, thereby facilitating ion diffusion and charge transfer in carbon aerogel composites [57]. Wang et al. [58] constructed a P-doped SiO_x_/graphene aerogel composite anode, in which P mainly existed in the form of P–C and P–O bonds and generated additional structural defects (Figure 6). Compared with the undoped sample, P-SiO_x_@GA (graphene aerogel) exhibited lower charge-transfer resistance and faster Li^+^ diffusion kinetics, indicating that phosphorus doping can improve interfacial transport between SiO_x_ and the conductive aerogel framework. Together with the continuous electron pathways and volume-buffering space provided by the three-dimensional graphene aerogel, this interfacially regulated composite structure effectively enhanced the rate capability and cycling stability of the SiO_x_ anode.

Boron doping in carbon aerogels is mainly associated with framework stabilization and interfacial-polarity regulation. The electron-deficient nature of boron atoms can induce local charge redistribution in the carbon framework, thereby enhancing electrode surface polarity and ion adsorption capability [59]. Zhao et al. [60] modified phenolic resin-derived carbon aerogels with boric acid. During the sol–gel process, boric acid participated in network construction and formed stable B–O bonds, improving the thermal stability and carbonization yield of the aerogels. The introduction of boric acid also reduced particle size, compacted the network structure, and improved electrode wettability and electrolyte-transport efficiency. These results indicate that boron modification is most valuable when framework retention and accessible-interface utilization are improved at the same time.

Sulfur doping more strongly affects surface polarity and interfacial binding states, thereby enhancing the chemical anchoring of reaction intermediates or active species [61]. After sulfur atoms are incorporated into the graphene aerogel framework, they can alter the local electronic structure through C–S bonds and strengthen the interfacial interaction between the carbon framework and active components. In lithium–sulfur systems, the porous carbon aerogel/sulfur composite cathode prepared by Lin et al. [62] mainly relied on the spatial confinement of sulfur and polysulfides within three-dimensional pore channels. Meanwhile, the interconnected carbon framework provided electron-transport pathways, while mesopores and macropores shortened ion-diffusion distances, thereby mitigating the shuttle effect and improving cycling stability. In this case, sulfur-containing sites mainly reinforce polar anchoring and interfacial charge transfer, while the porous aerogel network supplies spatial confinement and transport pathways.

We summarize the mechanisms, advantages, limitations, and regulatory roles of different dopant elements in carbon aerogels. The summary is presented in Table 4.

The effect of single heteroatom doping depends on the matching degree between the intrinsic characteristics of dopant atoms and the target energy storage process. Nitrogen, phosphorus, boron, and sulfur mainly emphasize active-site regulation, diffusion-environment optimization, local-polarity modulation, and interfacial anchoring enhancement, respectively. However, actual performance improvement is still constrained by pore accessibility, conductive-framework continuity, and doped-site stability. For single-element doping, the key comparison is whether a given dopant produces stable and accessible sites under the selected carbonization and electrochemical conditions. Site retention during heat treatment and pore accessibility after electrode fabrication should therefore be treated as core design criteria.

### 3.2. Multi-Heteroatom Doping

Multi-heteroatom doping is used when one dopant cannot simultaneously satisfy conductivity, ion affinity, and interfacial stability requirements. In carbon aerogels, N is commonly paired with S, B, or P because these combinations can redistribute charge, tune defect environments, and adjust ion-diffusion surroundings through different bonding configurations [63,64].

**Figure 7:**
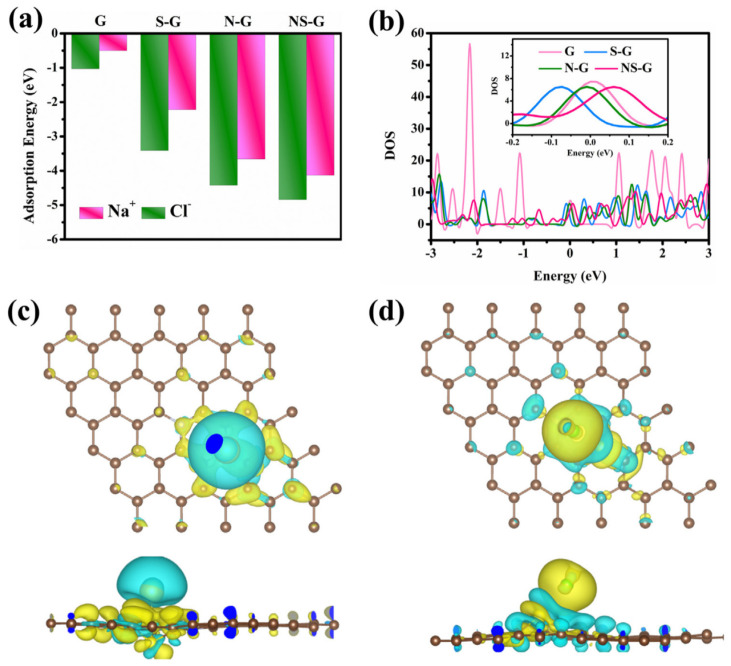
(**a**) Adsorption energies of Na^+^ and Cl^−^ ions for the various configurations; (**b**) density of states for G, S-G, N-G, and NS-G; differential charge density (top and side views) for NS-G with (**c**) Na^+^ and (**d**) Cl^−^ [66].

N/S co-doping enhances the energy storage performance of carbon aerogels through several coupled effects. These effects include defect generation, electronic-structure modulation, and improved interfacial wettability. N-containing configurations provide ion adsorption sites and promote electron transport. S-containing groups can enlarge interlayer spacing, induce structural defects, and enhance surface polarity through bonding states such as C–S and C–S–C. Gu et al. [65] investigated the effect of heteroatom configurations on lithium-storage behavior in N/S co-doped graphene aerogels by adjusting the calcination temperature and thiourea dosage. The results showed that the electrode performance was not solely determined by the total N/S content, but was more strongly dependent on the relative proportions of pyridinic N, graphitic N, C–S–C/C–S bonds, and oxygenated sulfur functional groups. Appropriate calcination removed unstable C–SO_3_–C and C–SO_2_–C groups, thereby reducing electrolyte decomposition and irreversible side reactions, while retaining relatively stable C–S–C/C–S sites to maintain lithium-storage activity. Increasing the thiourea dosage helped increase the pyridinic N content and create more active sites, but the decrease in graphitic N could weaken electronic transport. Moreover, the rate capability and cycling performance did not continuously improve with increasing thiourea dosage. These results indicate that heteroatom doping in carbon aerogels has an effective regulation window. Excessive precursor dosage or abundant unstable oxygenated sulfur functional groups may limit battery performance by reducing electronic conductivity, intensifying interfacial side reactions, and weakening structural/interfacial stability. Liu et al. [66] constructed N/S co-doped composite carbon aerogels using konjac glucomannan, fucoidan, and nitrogen-enriched carbon nanotubes. In this system, fucoidan served as the sulfur source and stabilized the polysaccharide framework, while N-CNTs provided the nitrogen source and a continuous conductive network. The optimized sample integrated high specific surface area, a hierarchical pore structure, and favorable wettability. DFT calculations showed that N/S co-doping induced charge redistribution and enhanced the adsorption capability toward Na^+^ and Cl^−^, as evidenced by the adsorption-energy, density-of-states, and differential charge density results in Figure 7.

N/B co-doping in aerogel-derived carbon materials mainly enhances charge transport and ion adsorption through the coupled effects of complementary electronic-structure modulation, interfacial-polarity regulation, and defect-site construction. Xiao et al. [67] prepared N/B co-doped carbon aerogels using cellulose-based aerogels as precursors through in situ N/B doping, carbonization, and activation with potassium citrate. The aerogel-derived framework provided hierarchical pore channels and continuous transport pathways, while N/B sites induced local charge redistribution and strengthened interfacial adsorption capability. This study indicates that the coupling between N/B co-doping and hierarchical pore architecture contributes to improving accessible-interface utilization and interfacial reaction kinetics in carbon aerogels.

N/P co-doping in carbon aerogels is mainly used to simultaneously regulate active-site chemistry and the ion-diffusion environment. Nitrogen atoms can modulate local charge distribution and form active sites such as pyridinic N and pyrrolic N. Phosphorus atoms have a larger atomic radius, and their incorporation into the carbon framework readily induces local distortion, interlayer-spacing expansion, and defect generation, thereby providing more favorable space for ion insertion and migration. Yu et al. [68] prepared cellulose-based carbon aerogels using urea phosphate as the N/P source. They found that a higher phosphorus-source content promoted the conversion of pyrrolic N to pyridinic N. Unstable oxygen-containing functional groups were also reduced. Accordingly, N/P co-doping is useful when active-site formation needs to be coupled with expanded diffusion space and reduced interfacial resistance.

Dual heteroatom doping can address specific limiting factors, such as interfacial adsorption, charge transport, or ion diffusion. In practical energy storage electrodes, however, these processes usually occur at the same time. They are also strongly coupled with pore accessibility and framework continuity. When only two dopant elements are introduced, the regulatory dimension may still be insufficient for complex electrochemical processes. Triple-element doping is therefore introduced as a broader regulation route, especially when pore transport, defect chemistry, and surface affinity must be adjusted simultaneously.

Compared with dual-element doping, N/P/S triple-element doping can simultaneously regulate the pore architecture, defect density, and interfacial chemistry of carbon aerogels. Jin et al. [69] prepared N/P/S co-doped coal-derived hierarchical porous carbon aerogels using coal as the precursor through modified sol–gel processing, freeze-drying, and carbonization. The introduction of N, P, and S altered the morphology of the aerogel framework, leading to a crosslinked, wrinkled honeycomb-like structure and further increasing pore-channel openness and structural defect density. XPS results showed that pyridinic N, pyrrolic N, C–S-related structures, and P–C/P–O bonds jointly participated in interfacial regulation. Among them, nitrogen species contributed to surface-polarity and electronic-structure modulation, sulfur species enhanced interfacial affinity and adsorption behavior, and phosphorus species induced framework distortion and defect generation. This study indicates that the performance enhancement of carbon aerogels by N/P/S triple-element doping mainly arises from the coupled effects of hierarchical pore-mediated mass transport, defect-site regulation, and interfacial-chemistry optimization, rather than a simple increase in total dopant content.

Multi-heteroatom doping research should shift from pursuing high total dopant content to constructing efficient, stable, and spatially accessible active sites. The dopant type, bonding configuration, relative content, and spatial distribution should be carefully considered. The doping process should not cause pore blockage, framework collapse, or damage to the continuous conductive network.

### 3.3. Summary

Heteroatom doping links pore-mediated transport, electronic regulation, and interfacial reactions in carbon aerogels. Single-element doping is useful for defining the functional boundary of a specific dopant, whereas multi-element doping enables more complex cooperation among charge regulation, defect construction, and interfacial affinity. However, higher dopant introduction does not necessarily lead to better electrochemical behavior. Excessive precursor dosage or unstable functional groups may disrupt conductive continuity and promote parasitic reactions. Future work should move from simply introducing dopants to identifying whether the doped sites remain effective under practical electrode conditions.

## 4. Alkali-Metal-Ion Batteries

In alkali-metal-ion batteries, carbon aerogels can serve as standalone anode materials. They can also act as conductive supports and stress-buffering frameworks for high-capacity active components [12]. Li^+^, Na^+^, and K^+^ differ in ionic radius, insertion thermodynamics, and diffusion behavior. Different requirements are therefore imposed on the pore-size distribution and framework stability of carbon aerogels. Based on these differences, the following sections discuss the applications of carbon aerogels in lithium-, sodium-, and potassium-ion batteries. Particular attention is paid to their roles in charge transport, ion diffusion, volume-stress accommodation, and interfacial stabilization.

### 4.1. Lithium-Ion Batteries

In lithium-ion batteries, carbon aerogels are commonly employed as conductive scaffolds and stress-buffering matrices for high-capacity active components. Their interconnected three-dimensional networks can maintain continuous charge transport, while hierarchical pore channels facilitate electrolyte infiltration, shorten Li^+^ diffusion pathways, and provide void space for volume-variation accommodation [70,71,72]. In addition, surface coating, heteroatom doping, and interfacial regulation can help stabilize the electrode/electrolyte interface and mitigate repeated SEI reconstruction [73].

Loading high-capacity active components onto the three-dimensional conductive framework of carbon aerogels is a common strategy for increasing the anode capacity of lithium-ion batteries. Such an architecture can compensate for the limited capacity of pure carbon materials, while dispersing reaction-induced stress and maintaining electronic contact through the interconnected porous network. Zhao et al. [74] developed a SnO_2_/SnS_2_ quantum-dot-decorated sulfur-doped graphene aerogel as an anode for lithium-ion batteries. The material retained a continuous three-dimensional porous graphene framework while uniformly dispersing 2–6 nm SnO_2_/SnS_2_ quantum dots throughout the conductive network. The interconnected porous architecture facilitated electrolyte penetration and Li^+^ transport, while also providing buffer space for volume changes during cycling. In addition, the homogeneous distribution of quantum dots helped suppress particle aggregation and reduce localized mechanical stress. From the perspective of interfacial chemistry, the abundant pores and enlarged surface area exposed more electrochemically active sites and enhanced surface-controlled lithium storage. However, the increased electrode–electrolyte contact area could also accelerate electrolyte decomposition and SEI formation during the initial cycle, resulting in irreversible lithium consumption. Sulfur doping introduced polar surface sites and modified the local electronic structure of the graphene framework, which strengthened Li^+^ affinity and promoted interfacial charge-transfer kinetics. The relatively high initial Coulombic efficiency of 83.2% suggests that, although the porous structure and defect-rich surface inevitably contributed to SEI formation, the sulfur-induced interfacial regulation and uniform distribution of active nanoparticles improved the reversibility of interfacial reactions. These results indicate that SEI evolution and irreversible capacity loss are jointly governed by accessible surface area, pore architecture, defect density, and sulfur-induced surface polarity.

**Figure 8:**
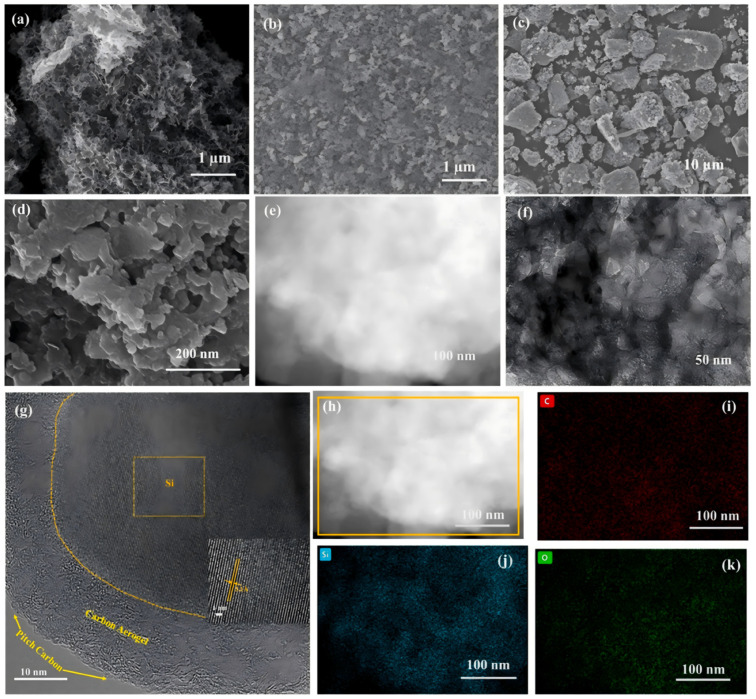
SEM images of (**a**) carbon aerogel, (**b**) silicon nanoparticles, and (**c**,**d**) SCAP-22; (**e**) TEM image of SCAP-22; (**f**) TEM image of SCAP-22 after eliminating silicon; (**g**) HRTEM image of SCAP-22; (**h**–**k**) element mapping of SCAP-22 [75].

For Si/C anodes, the effective design of carbon aerogels should move from single framework support toward multilevel carbon-interface synergy, in which conductive frameworks, buffering space, and outer carbon coatings jointly suppress Si volume variation and interfacial side reactions. Saleem et al. [75] constructed a sodium alginate-derived Si/carbon aerogel composite anode through freeze-drying, wet ball milling, and carbonization. Representative SEM, TEM, HRTEM, and elemental mapping images of the composite are shown in Figure 8. In this route, sodium alginate first formed a porous aerogel precursor with Si nanoparticles, after which coal pitch was introduced through wet ball milling and carbonized at 800 °C to form a pitch-derived carbon layer, ultimately yielding pitch-coated silicon-coated carbon aerogel (SCAP-x) composites, where x denotes the coal pitch content. In this structure, the carbon aerogel framework provided dispersion space and continuous conductive pathways for Si nanoparticles, while the pitch-derived carbon layer further strengthened the interfacial connection between Si and the carbon framework and reduced direct contact between Si and the electrolyte. The optimized SCAP-22 retained a reversible capacity of 541 mAh g^−1^ after 1000 cycles at 1 A g^−1^ and maintained 505 mAh g^−1^ at 2 A g^−1^. Compared with single carbon framework support, this work highlights the functional division between the carbon aerogel skeleton and the outer carbon coating. The carbon aerogel mainly contributes to Si dispersion, conductive support, and volume buffering, whereas the pitch-derived carbon layer reinforces interfacial protection and SEI stabilization. Therefore, for Si-containing composite anodes, carbon aerogel design should not only pursue high porosity, but also regulate the balance among outer carbon-coating thickness, Si exposure degree, and Li^+^ transport resistance, thereby simultaneously maintaining ion accessibility and interfacial stability.

In addition to Si-containing composite systems, carbon aerogels can also be used in metal-compound conversion-type anodes to alleviate volume variation, particle aggregation, and conductive failure during conversion reactions. Jia et al. [76] prepared a binary transition metal selenide coated with a carbon aerogel veil, NiCoSe@GC. In this work, sodium alginate, melamine, CoCl_2_·6H_2_O, and NiCl_2_·6H_2_O were used as raw materials. An aerogel containing Ni and Co precursors was first obtained by freeze-drying, followed by high-temperature calcination to form NiCo@GC and subsequent selenization to produce NiCoSe@GC. The resulting NiCoSe particles were confined in situ within the N-doped carbon aerogel framework. The carbon aerogel provided continuous charge-transport pathways and porous ion-diffusion channels, while accommodating the volume variation of metal selenides during lithiation and delithiation. NiCoSe@GC delivered an initial discharge capacity of 941.1 mAh g^−1^ in lithium-ion batteries and retained 768.5 mAh g^−1^ after 700 cycles at 1 A g^−1^, markedly outperforming the control sample without the carbon aerogel structure. These results indicate that the confinement effect of carbon aerogels can effectively improve active-phase dispersion, capacity retention, and interfacial stability in conversion-type composite anodes.

**Figure 9:**
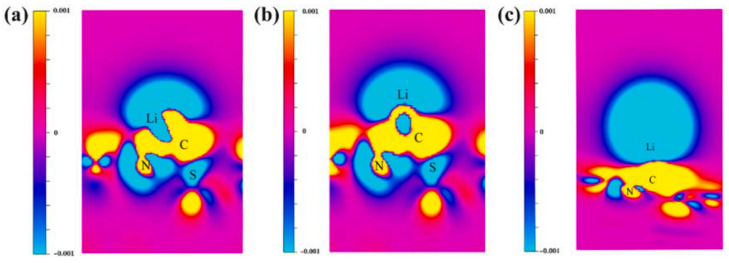
2D charge-density difference maps of a Li atom adsorbed on doped graphene at different adsorption sites: (**a**) H site, (**b**) B site, and (**c**) T site [77].

Researchers have further examined the mechanism behind the improved Li storage performance of carbon aerogel-based electrodes by correlating pore hierarchy and heteroatom-induced active sites with Li adsorption and interfacial charge transfer. Feng et al. [77] designed N/S dual-doped porous graphene aerogels/natural graphite composites as anodes for lithium-ion batteries. Natural graphite particles were embedded in a three-dimensional porous graphene aerogel network, which provided continuous electron-transfer pathways and electrolyte-accessible ion channels. The micro/mesoporous structure, with pore-size distributions around 3.8 and 12 nm, supplied additional Li storage sites and promoted electrolyte penetration, while N/S dual doping increased defect density and introduced pyridinic-N, pyrrolic-N, C–S–C, and C=S configurations. Owing to this structural and chemical coupling, the electrode delivered 531 mAh g^−1^ at 0.1 C, retained 277 mAh g^−1^ at 2 C, and maintained 474.7 mAh g^−1^ after 200 cycles at 0.5 C. DFT calculations further revealed that N/S co-doping strengthened Li adsorption and regulated interfacial charge transfer (Figure 9). The Li adsorption energy at the H site of graphene aerogels was −1.187 eV, whereas that at the defect-center H site of N/S dual-doped porous graphene aerogels/natural graphite composites (N,S-pGA/NG) decreased to −2.455 eV. Differential charge density analysis showed local charge redistribution around N and S atoms, and projected density-of-states (PDOS) results indicated increased electronic states near the Fermi level. These results link heteroatom configuration with Li adsorption, charge-transfer kinetics, high-rate capability, and cycling stability.

In lithium-ion batteries, carbon aerogels mainly contribute to the stable loading of high-capacity active components, maintenance of charge-transport pathways, accommodation of volume variation, and suppression of interfacial side reactions. Different composite systems impose different structural requirements on carbon aerogels. Si-containing composite anodes rely more strongly on multilevel carbon coating, stable Si–carbon framework connection, and long-term SEI stability, whereas conversion-type composite anodes based on metal oxides, sulfides, or selenides place greater emphasis on active-phase confinement, particle dispersion, and conductive-network continuity. Future designs should further strengthen the interaction between active components and carbon frameworks. Long-term SEI evolution should also be regulated, and conductive-network continuity must be maintained during cycling. Such optimization can mitigate volume expansion, particle isolation, and interfacial side reactions. Carbon aerogel-based composite anodes can achieve a more stable balance among high capacity, rate capability, and cycle life.

### 4.2. Sodium-Ion Batteries

Sodium-ion battery anodes are strongly limited by the large ionic radius of Na^+^. Sluggish diffusion kinetics and cycling-induced structural stress further restrict their electrochemical performance [78]. For carbon aerogels, their function should not be understood simply as providing high specific surface area. More attention should be paid to the coupled roles of open pore channels, enlarged interlayer spacing, continuous conductive frameworks, and volume-accommodation space in sodium storage. Existing studies can generally be classified into two routes. In the first route, carbon aerogels are directly used as standalone sodium-storage anodes. Their porous structures, inherited from biomass or precursor frameworks, shorten Na^+^ transport pathways. In the second route, carbon aerogels serve as conductive scaffolds and stress-buffering matrices for high-capacity active components. Capacity output and cycling stability can therefore be improved [79].

For intrinsic carbon aerogel anodes, sodium-storage performance is not just governed by specific surface area. It is more closely related to the balance among interlayer spacing, pore-channel connectivity, and carbonization degree. Yang et al. [80] used bagasse as the carbon source. Biomass-derived carbon aerogels were prepared through hydrothermal treatment, freeze-drying, and carbonization for sodium-ion battery anodes. Bagasse provided a honeycomb porous carbon framework. Micropores provided storage sites for Na^+^. Mesopores and macropores promoted electrolyte infiltration and ion transport. The optimized sugarcane bagasse-derived carbon aerogel carbonized at 1200 °C combined suitable interlayer spacing, a hierarchical pore architecture, and favorable electrical conductivity, delivering an initial discharge capacity of 380 mAh g^−1^ at 50 mA g^−1^ and retaining 248 mAh g^−1^ after 50 cycles. This study indicates that, when biomass-derived carbon aerogels are used as sodium-storage anodes, structural retention, interlayer environment, and carbonization temperature should be carefully controlled to avoid pore-structure degradation and interlayer-spacing contraction caused by excessive carbonization.

In composite carbon aerogel anodes, the primary role of the three-dimensional aerogel framework is to stabilize high-capacity active phases and provide charge-transport pathways and volume-accommodation space for conversion reactions. Dong et al. [81] constructed a MoS_2_/N-doped graphene aerogel (MoS_2_/NGA) composite anode for sodium-ion batteries. The material consists of MoS_2_ nanosheets anchored on a three-dimensional interconnected N-doped graphene aerogel network, with a specific surface area of 114 m^2^ g^−1^, a pore volume of 0.44 cm^3^ g^−1^, a dominant pore size of approximately 3.7 nm, and additional micropores. The meso/microporous structure promotes electrolyte penetration into the aerogel network and exposes MoS_2_/NGA heterointerfaces, graphene edges, and N-doped sites during the first discharge. These open interfaces facilitate Na^+^ insertion into MoS_2_ layers, MoS_2_ conversion reactions, and surface-controlled sodium storage, but they also enlarge the region for electrolyte reduction. In the CV curves, the cathodic peak at about 0.33 V appears only in the first cycle and is attributed to the formation of Na_x_MoS_2_ together with SEI formation. The first discharge and charge capacities are approximately 1080 and 530 mAh g^−1^, giving a low initial Coulombic efficiency of 48.5%, which indicates substantial irreversible Na consumption during SEI growth and surface side reactions. N doping further regulates the interfacial sodium-storage process. XPS analysis confirms the presence of pyridinic N, pyrrolic N, and graphitic N in MoS_2_/NGA. Pyridinic and pyrrolic N located at graphene edges enhance Na^+^ adsorption, whereas graphitic N improves electronic transport. EIS analysis shows that the charge-transfer resistance decreases during charge/discharge, indicating improved electron transfer and solid-state Na^+^ diffusion through the aerogel network and MoS_2_/NGA interfaces. Scan-rate-dependent CV analysis gives b values of 0.89 and 0.91 for the reduction and oxidation peaks, respectively, showing a dominant surface-controlled process. The capacitive contribution reaches 76.1% at 0.2 mV s^−1^. Post-cycling SEM/EDS further shows no obvious cracking after 80 cycles and a relatively uniform Na distribution, suggesting that the three-dimensional aerogel network maintains interfacial contact after SEI stabilization. These results indicate that the initial irreversible capacity loss mainly originates from highly exposed porous and N-defect-rich interfaces, while the stabilized capacity is supported by interfacial pseudocapacitive sodium storage, MoS_2_ conversion reactions, and continuous Na^+^/electron transport through the aerogel network.

**Figure 10:**
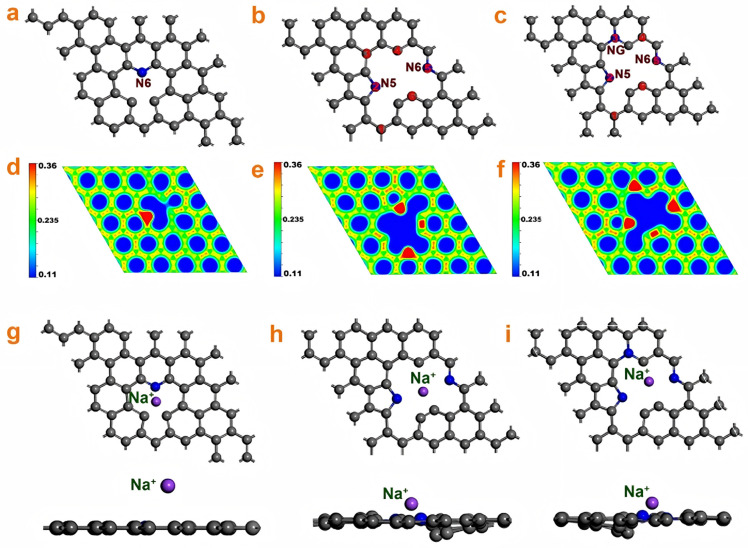
DFT-optimized structures of N-doped graphene for (**a**) pyridinic N (N6), (**b**) pyridinic N/pyrrolic N defects (N6-N5-1), and (**c**) N6-N5-NG-1. (**d**–**f**) Electron densities corresponding to (**a**–**c**), with the blue and red regions corresponding to electron-rich and electron-deficient regions, respectively. Top and side views of optimized structures of Na+ adsorbed on N-doped graphene for (**g**) N6, (**h**) N6-N5-1, and (**i**) N6-N5-NG-1 [82].

Researchers further combined DFT calculations to probe the sodium-storage mechanism of N-doped carbon aerogels. Lu et al. [82] prepared N-doped carbon nanofiber aerogels from chitin nanofibers through carbonization in a N_2_/NH_3_ mixed atmosphere followed by KOH activation, and evaluated them as anodes for sodium-ion batteries. After activation, the aerogel retained a continuous three-dimensional nanofiber network while developing abundant micro/mesopores. Its BET surface area increased from 65.27 to 746.16 m^2^ g^−1^, and the micropore volume rose from 0.004 to 0.111 cm^3^ g^−1^, which facilitated electrolyte infiltration, shortened Na^+^ diffusion pathways, and exposed more accessible storage sites. Meanwhile, the N content decreased from 12.2 to 5.3 at.%, indicating partial removal of structural N and formation of vacancy/edge defects. As a result, the material delivered 178–179 mAh g^−1^ at 5 A g^−1^ and maintained over 238 mAh g^−1^ after 7000 cycles at 0.5 A g^−1^. DFT calculations further showed that isolated N sites provided limited Na adsorption, as shown in Figure 10. Among the DFT models, the defective N-doped graphene configuration containing pyridinic N, pyrrolic N, and graphitic N (N6-N5-NG-1) exhibited a much stronger Na adsorption energy of −1.67 eV. Therefore, the enhanced sodium-storage performance mainly arises from the coupling of hierarchical pores, N-induced charge redistribution, and defect-dominated adsorption/pseudocapacitive sites. However, the low initial Coulombic efficiency of 24.3% indicates that excessive porosity and defects may aggravate irreversible SEI formation.

The two types of studies reveal distinct functional roles of carbon aerogels in sodium-ion batteries. Intrinsic carbon aerogels mainly regulate Na^+^ insertion and diffusion through interlayer spacing, pore architecture, and carbonization degree. Their advantages include structural simplicity, relatively low cost, and compatibility with sustainable precursors. However, their capacity is still limited by the intrinsic sodium-storage capability of the carbon framework. By contrast, high-capacity active-phase/aerogel composites can markedly increase capacity output. Volume variation of the active phase can also be accommodated by the three-dimensional framework. However, low initial Coulombic efficiency, continuous SEI evolution, and long-term active-phase stability remain critical issues. Future designs of carbon aerogels for sodium-ion batteries should avoid the simple pursuit of high specific surface area. More attention should be paid to controllable interlayer structures, stable pore-channel connectivity, suppressed interfacial side reactions, and stabilized active-phase loading. Such efforts are expected to improve effective sodium-storage capability under long-cycle and high-rate conditions.

### 4.3. Potassium-Ion Batteries

Potassium-ion batteries offer several advantages, such as abundant potassium resources, relatively low cost, and a low redox potential. These features make them promising candidates for large-scale energy storage applications. Compared with Li^+^ and Na^+^, K^+^ has a larger ionic radius. A larger ionic radius increases the energy barriers for insertion and migration, leading to limited ion-diffusion kinetics [83]. Repeated K^+^ insertion/extraction within carbon interlayers can induce more severe structural strain [84].

Heteroatom doping can regulate the potassium storage performance of carbon aerogels. K^+^ adsorption, charge-transfer efficiency, and interfacial wettability can be affected by doped sites. Xie et al. [85] compared the potassium storage behavior of carbon aerogels with single heteroatom doping (O, N, or B) and co-doping. They found that O/N co-doped carbon aerogels exhibited favorable K^+^ adsorption energy (−1.62 eV) and a low diffusion energy barrier (0.12 eV). Although O/N/B ternary doping introduced more dopant atoms, its overall performance was inferior to that of the O/N co-doped system. This difference was mainly caused by the lower stability of B sites and reduced surface wettability. This study indicates that doping design for potassium-ion batteries should prioritize effective-site stability, interfacial wettability, and electronic-structure matching. Increasing the number of dopant elements alone does not necessarily improve electrochemical performance.

Doped sites can also couple with electrolyte chemistry to influence SEI composition and interfacial stability. Gao et al. [86] showed that N/P dual-doped graphene aerogels more readily formed a KF-rich inorganic-dominated SEI in a potassium bis(fluorosulfonyl)imide electrolyte, whereas an organic–inorganic hybrid SEI was formed in a KPF_6_ electrolyte. The KF-rich interface possessed higher chemical stability and mechanical flexibility, increasing the Coulombic efficiency from 85.09% to 97.56%. This result suggests that doped sites in potassium-ion batteries affect not only K^+^ adsorption and diffusion, but also electrolyte decomposition pathways and SEI composition. For carbon aerogel anodes, doping design should therefore be considered together with the electrolyte system to stabilize the electrode interface during repeated insertion/extraction of large-radius K^+^.

**Figure 11:**
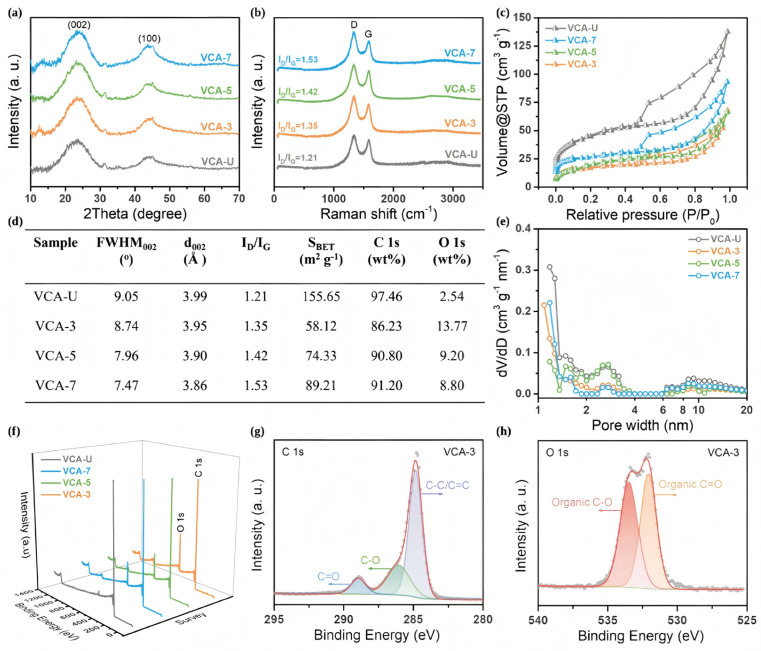
(**a**) XRD patterns, (**b**) Raman spectra, (**c**) nitrogen adsorption–desorption isotherms, and (**e**) pore-size distributions. (**d**) Summary of structural and surface elemental parameters, and (**f**) full XPS survey spectra of VCA-U, −3, −5, and −7; VCA-U refers to the vertically aligned carbon aerogel prepared by uncontrolled freezing. High-resolution XPS spectra of (**g**) C 1s and (**h**) O 1s core levels for VCA^−3^ (a.u. = arbitrary units) [87].

Structure-oriented design can improve K^+^ storage through coordinated pore-channel orientation and interlayer-environment regulation. Rather than simply increasing porosity, oriented carbon aerogels can shorten ion-transport pathways and provide more suitable space for K^+^ intercalation through interlayer-spacing regulation. Wang et al. [87] constructed oriented carbon aerogels using a unidirectional ice-templating method and regulated their pore-channel orientation and interlayer spacing by adjusting the cooling rate. As shown in Figure 11, XRD, Raman, nitrogen adsorption–desorption, pore-size distribution, and XPS analyses were used to reveal the structural evolution, pore characteristics, and surface chemistry of vertically aligned carbon aerogel (VCA) samples prepared at different cooling rates. The results showed that different structural parameters corresponded to different storage behaviors. Vertically aligned carbon aerogel prepared at a cooling rate of 3 K min^−1^ (VCA-3) was more favorable for surface adsorption storage of Na^+^, whereas VCA-5, with a moderate degree of graphitization and enlarged interlayer spacing, was more suitable for reversible K^+^ intercalation and retained 82.7% of its capacity after 1000 cycles. This result indicates that carbon aerogels for potassium-ion batteries should not simply pursue high porosity, but should balance the degree of framework ordering, interlayer spacing, and oriented transport channels. Excessive disorder or ineffective porosity may instead weaken the long-term stability of potassium storage.

For high-capacity conversion-type anodes, carbon aerogels can serve as conductive scaffolds and stress-buffering matrices, thereby improving the structural reversibility of active components. To address nanosheet stacking and structural collapse of VSe_2_ during cycling, Feng et al. [88] constructed a carbon nanobelt aerogel coating structure. The three-dimensional crosslinked network restricted VSe_2_ aggregation, accommodated volume variation during potassiation/depotassiation, and maintained continuous charge transport. Oxygen doping further enhanced interfacial K^+^ adsorption and lowered the diffusion energy barrier, thereby improving reaction kinetics. This study indicates that, in conversion-type potassium-ion battery anodes, the role of carbon aerogels is not limited to conductive support, but also includes active-phase confinement, stress buffering, and interfacial adsorption regulation. The aerogel network better maintains active-phase dispersion. It also improves long-term cycling stability compared with conventional carbon coating.

**Figure 12:**
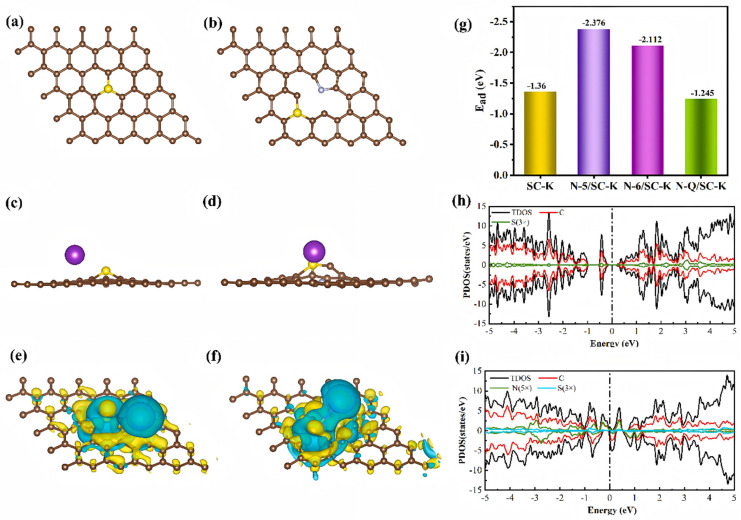
Simulation for several structures theoretically: (**a**) S-doped graphene, (**b**) pyrrole N/S-doped graphene, (**c**,**d**) The most stable confgurations of K atom adsorbed on S-doped graphene and pyrrole N/S-doped graphene, (**e**,**f**) differential charge density, (**g**) Adsorption energy of various models, (**h**,**i**) the density of states of S-doped graphene and pyrrole N/S-doped graphene.

Researchers further combined DFT calculations with electrochemical kinetic analysis. Qi et al. [89] prepared N/S co-doped mesh-like porous carbon using sodium polyacrylate as the carbon source and Na_2_S_2_O_3_/NaNO_3_ as S and N sources through freeze-drying-assisted carbonization. The optimized N/S co-doped porous carbon carbonized at 700 °C (NSC-700) exhibited a crosslinked hierarchical porous structure with a surface area of 2160 m^2^ g^−1^ and a pore volume of 2.18 cm^3^ g^−1^, providing open channels for electrolyte infiltration and K^+^ transport. N/S co-doping increased structural disorder and introduced pyridinic N, pyrrolic N, graphitic N, and C–S bonding sites, which supplied additional adsorption/pseudocapacitive storage sites. The electrode retained 274.8 mAh g^−1^ after 200 cycles at 0.05 A g^−1^ and showed better rate capability than the S-doped counterpart. Kinetic analysis gave a b value of 0.86, and the capacitive contribution increased from 56.7% to 88.8% as the scan rate increased from 0.1 to 1.5 mV s^−1^, indicating a dominant surface-controlled process. DFT calculations further compared K adsorption and migration on S-doped graphene and different N/S co-doped models (Figure 12). The K adsorption energy of S-doped graphene was −1.36 eV, whereas pyrrolic N/S co-doping showed a much stronger adsorption energy of −2.376 eV, higher than those of graphitic N/S and pyridinic N/S configurations. Differential charge density analysis revealed electron transfer from K to the doped carbon matrix, while N/S co-doping generated more polar electron-deficient active centers. The increased density of states near the Fermi level further suggests improved charge-transfer capability. Notably, stronger K adsorption also raises the local migration barrier, from 0.124 eV on S-doped graphene to 0.727 eV on pyrrolic N/S-doped graphene. Thus, the high-rate performance arises from the coupling of shortened macroscopic diffusion pathways, enhanced K^+^ adsorption/electron transfer at N/S defect sites, and capacitive potassium storage, rather than from a lower atomic migration barrier alone.

Potassium-ion batteries place higher requirements on the collapse resistance of aerogel frameworks and the continuity of charge transport pathways under high-rate conditions because of the larger radius of K^+^. High specific surface area or high defect density alone is far from sufficient for potassium-ion batteries.

Because the ion size, storage mechanism, interfacial stability, and structural stress differ markedly among lithium-, sodium-, and potassium-ion batteries, Table 5 summarizes the corresponding structural requirements, functional roles, and design priorities of carbon aerogels in these electrochemical energy storage systems.

### 4.4. Summary

From the perspective of storage mechanisms, the roles of carbon aerogels in lithium-, sodium-, and potassium-ion batteries should be compared through adsorption, pore filling, defect storage, and interlayer insertion. Li^+^ has a smaller ionic radius and can more readily undergo interlayer insertion in ordered or locally graphitized carbon domains. Therefore, in lithium-ion systems, carbon aerogels mainly improve rate capability and cycling stability by providing continuous conductive networks, pore-buffering space, and stable interfaces, while surface adsorption, pore-wall adsorption, and defect storage offer additional capacity. Na^+^ has a larger radius, and reversible insertion into conventional carbon layers is more restricted. Its storage behavior therefore relies more on surface/edge adsorption, heteroatom-induced polar sites, defect storage, and nanopore filling. In sodium-ion systems, sloping-region capacity is often associated with defect and surface adsorption, whereas the low-voltage plateau is more closely related to limited interlayer insertion, closed-pore/nanopore filling, and possible Na-cluster storage. K^+^ has an even larger radius and slower insertion/diffusion kinetics, placing higher demands on interlayer spacing, pore connectivity, and structural stability. Enlarged interlayer spacing can relieve the structural strain caused by K^+^ insertion, meso/macroporous channels improve electrolyte infiltration and ion transport, and N-, S-, P-, or B-induced defects and polar sites strengthen K^+^ adsorption, increase pseudocapacitive contribution, and sustain capacity under high current densities. Overall, adsorption, pore filling, defect storage, and interlayer insertion are coupled in carbon aerogels and are jointly regulated by ion size, pore-size distribution, closed-pore fraction, carbon-layer ordering, defect density, and surface chemistry. Thus, lithium-ion systems emphasize conductive continuity, interlayer insertion, and interfacial stability, and sodium-ion systems require a balance among defect adsorption, pore filling, and limited insertion, while potassium-ion systems depend more strongly on enlarged interlayer spacing, open channels, and surface-controlled storage.

Electrochemical performance is a direct criterion for evaluating the effectiveness of carbon aerogel-based electrode design. However, the reported results are strongly affected by the battery system, active material composition, current density, cycling protocol, and cell configuration. Therefore, representative capacity, half-cell cycling performance, full-cell cycling performance, and ICE values are summarized in Table 6 to provide a clearer comparison of carbon aerogel-based anodes in different alkali-metal-ion batteries.

These mechanistic differences also mean that electrochemical performance should be interpreted together with testing conditions. For carbon aerogels with high surface area, open pores, and abundant defects, electrolyte decomposition, SEI growth, irreversible ion consumption, and ion trapping can strongly affect the initial Coulombic efficiency and long-term capacity retention. Metal-electrode half-cells are useful for preliminary screening, but they may not directly reflect practical device behavior. The metal counter electrode provides a large excess of active-ion inventory, which can partially mask capacity losses caused by SEI formation, irreversible ion consumption, and ion trapping [90]. Meanwhile, metal stripping/plating generates pronounced overpotentials and high-surface-area metal deposits, so the measured cell voltage does not necessarily reflect the true potential of the working electrode. This may distort the interpretation of capacity, rate capability, and cycling degradation. For high-surface-area carbon aerogels or alloying-type composite anodes, open pores, defect sites, and highly reactive interfaces readily promote electrolyte decomposition and SEI growth [91]. However, excess active-ion supply and the commonly used excess electrolyte in metal-electrode cells may weaken the apparent influence of these parasitic reactions on capacity fading. Excess electrolyte can also alter long-term cycling behavior, gas evolution, and the accumulation of interfacial decomposition products, causing the results to deviate from practical lean-electrolyte conditions [92]. Low mass loading refers to a small amount of active material per electrode area, which shortens ion-diffusion pathways, improves electrolyte wetting, and reduces polarization, thereby making high-rate and long-cycle performance easier to obtain than in practical thick electrodes. High specific capacity or long cycling stability obtained from low-mass-loading electrodes, excess electrolyte, and metal-electrode cells may overestimate the practical value of the material [93]. More reliable evaluation should combine practical mass loading, controlled electrolyte amount, capacity-balanced full cells, and, when necessary, three-electrode diagnostics to distinguish intrinsic material behavior from performance amplification caused by non-practical testing conditions.

These considerations further indicate that the design of carbon aerogel electrodes should be based on the correspondence among storage mechanisms, structural regulation, and practical testing conditions. A general design principle can therefore be established from the dominant limitation of each battery system. The pore architecture and heteroatom doping strategy should be adjusted according to the ion-storage mechanism, required pore structure, and electrochemical activity of different batteries. Specifically, the initial pore network and framework connectivity should be regulated during sol–gelation by controlling catalyst type, catalyst dosage, R/C ratio, and pH. Alkaline catalysis is usually favorable for forming highly crosslinked and micropore-dominated networks, whereas acidic catalysis tends to generate hybrid micro/mesoporous structures. A higher catalyst concentration generally produces denser nucleation sites, smaller primary particles, and a more refined pore structure, while a lower catalyst concentration favors the formation of larger pores. During drying, pore retention and framework integrity should be prioritized, and supercritical drying, freeze-drying, or ambient-pressure drying should be selected according to structural requirements, cost, and scale-up feasibility. During carbonization, conductivity improvement, pore-structure stability, and active-site retention should be balanced. Activation should be controlled within a moderate range to increase accessible porosity and specific surface area while avoiding framework damage caused by excessive etching. Heteroatom doping should focus on stable bonding configurations, accessible active sites, and spatial distribution rather than simply increasing the total dopant content. Ultimately, carbon aerogel electrodes should integrate hierarchical pore structures, continuous conductive networks, stable doped sites, robust interfacial stability, and scalable processing compatibility to improve capacity, initial Coulombic efficiency, rate performance, and long-term cycling stability.

## 5. Industrial Applications and Challenges

The industrial exploration of carbon aerogel-related anode materials has initially concentrated on Si/C anodes for lithium-ion batteries. Silicon anodes possess high theoretical capacity, but they suffer from severe volume expansion, interfacial side reactions, repeated SEI reconstruction, and loss of electrical contact. Therefore, industrial development focuses on whether porous carbon frameworks can simultaneously provide sufficient silicon-loading space, volume-accommodation capacity, conductive-network continuity, and compatibility with electrode processing [94]. Publicly available information indicates that related routes mainly revolve around precursor selection, pore-architecture regulation, silicon incorporation, carbon coating, and scalable preparation. Among these factors, pore-structure stability, compaction density, batch-to-batch consistency, and process scale-up capability are key parameters determining the practical application value of carbon aerogel-derived Si/C anode materials [95].

Group14 Technologies’ SCC55^®^ (Group14 Technologies, Woodinville, Washington, USA, Si/C anode material) can be regarded as a representative Si/C anode route based on aerogel/xerogel chemistry-related porous carbon frameworks [96]. In this route, phenolic compounds, aldehydes, crosslinkers, and related precursors are used to construct carbon aerogel or xerogel frameworks with tunable pore structures, followed by gas-phase infiltration of silicon-containing precursors such as silane to generate amorphous nanosilicon within the porous framework [97,98]. According to publicly available information, its BAM-3 plant (Group14 Korea production facility) in Korea was put into operation in 2026, with a designed annual capacity of 2000 tons, and the product cycle life can reach 1500–3000 cycles [99]. This route indicates that the industrial value of aerogel-derived porous carbon frameworks mainly originates from the synergy among pore-channel designability, uniform nanosilicon deposition, conductive-framework retention, and continuous manufacturing.

Chinese enterprises are also advancing Si/C anode development through resin-derived porous carbon and aerogel-derived carbon framework routes. Jinan Shengquan (Jinan Shengquan Group Share-Holding Co., Ltd., Jinan, China) has established two porous carbon routes, namely phenolic resin-based and reconstructed resin-based systems [100]. In the phenolic resin-based route, resin is used as the precursor to prepare spherical porous carbon through pore-channel regulation and activation-induced pore formation. The reconstructed resin-based route uses biomass-refining byproducts as carbon sources, placing greater emphasis on raw-material cost reduction and batch-to-batch stability. According to publicly available information, its porous carbon production line was upgraded to 1000 tons per year in 2025, with an annual output reaching 741.98 tons [101]. Sinosteel Maanshan Institute of Mining Research (Sinosteel Maanshan Institute of Mining Research Co., Ltd., Ma’anshan, China) developed a resin-based carbon aerogel technology route. The institute reportedly achieved hundred ton-scale production in 2023, entered the batch supply stage in 2025, and built a carbon aerogel production line with an annual capacity of 300 tons [102].

Biomass-derived carbon aerogels are becoming an important option for the low-cost preparation of Si/C anodes. Wanhua Chemical (Wanhua Chemical Group Co., Ltd., Yantai, China) used natural polymers, such as carrageenan and cellulose esters, as carbon precursors. Flexible carbon aerogel-coated anodes were prepared through gel network construction and high-temperature carbonization [103]. This route integrates natural polymer gelation, carbon aerogel framework formation, and interfacial coating of silicon particles. It is expected to reduce precursor cost and provide a stress-buffering microenvironment for Si during cycling. For practical application, however, key issues such as compositional fluctuation of biomass feedstocks, pore-structure consistency, carbonization-induced shrinkage, coating uniformity, and batch-to-batch stability still need to be further addressed.

The commercialization challenges of carbon aerogel-derived Si/C anodes for lithium-ion batteries mainly lie in cost control, batch-to-batch stability, and long customer-validation cycles. For aerogel-derived porous carbon frameworks, pore-architecture controllability, uniform silicon deposition, compaction density, and batch consistency need to be achieved simultaneously. However, silane-based vapor deposition, interfacial carbon coating, and post-treatment processes further increase equipment investment and manufacturing cost. Before being introduced into mainstream cell systems, these materials must also be validated in terms of long-term cycling stability, safety, storage stability, and production-line compatibility, thereby meeting the requirements for large-scale application.

Compared with Si/C anodes for lithium-ion batteries, carbon aerogel-related routes in sodium-ion batteries are more closely directed toward precursor design and pore-structure regulation for hard-carbon anodes. Owing to the larger ionic radius of Na^+^, sodium-storage behavior depends more strongly on suitable interlayer spacing, stable pore-channel connectivity, and low-cost carbon sources. Therefore, industrial efforts have mainly focused on biomass-derived carbon, resin aerogel-derived hard-carbon precursors, and coal-based hard-carbon routes. Publicly available information shows that related strategies mainly revolve around raw-material cost, interlayer-structure regulation, fast-charging capability, and scalable preparation [104].

Wuxi Anna Energy Technology Co., Ltd. (Wuxi, China) has disclosed a biomass-derived N/O co-doped carbon aerogel anode route for sodium-ion batteries [105]. In this route, biomass sources such as sugarcane, pomelo peel, durian shell, and winter melon can be used as precursors, and carbon aerogel anode materials are obtained through aerogel construction, carbonization, and heteroatom doping. Patent data show that the material can deliver an initial discharge capacity of 625 mAh g^−1^ at 50 mA g^−1^ and a reversible capacity of 361 mAh g^−1^ after 50 cycles. Suzhou Yituolian International Trade Co., Ltd. (Suzhou, China) publicly sells phenolic aerogels, resin-based hard-carbon precursors, and hard carbon for sodium-ion battery anodes, representing a supply-side route extending from resin aerogels toward hard-carbon anode precursors [106]. Carbon aerogels show important industrial value for large-scale production in sodium-ion batteries because low-cost precursor selection and interlayer/pore-structure regulation can be achieved.

Biomass-, resin-, and coal-based precursors are widely available and low in cost for sodium-ion batteries. However, high and fluctuating ash and impurity contents can easily compromise the consistency of carbon aerogels between batches.

At present, potassium-ion batteries have not yet formed a clear enterprise-level technology route based on carbon aerogel anodes. Potassium-ion batteries are more likely to use commercial graphite anodes [107]. This is because K^+^ shows good reversible insertion/extraction behavior in graphite, which reduces the industrial demand for carbon aerogels [108]. The use of carbon aerogels in potassium-ion batteries is still largely limited to laboratory-scale research.

Overall, the industrialization of carbon aerogel-related anode materials should not be evaluated only by production capacity or cycling performance. Precursor cost, production yield, batch-to-batch consistency, drying and carbonization energy consumption, environmental management, and volumetric electrode performance should also be considered. Resin-derived routes offer stable raw-material supply, tunable pore structures, and potential compatibility with continuous production, but phenolic condensation, solvent exchange, drying shrinkage, carbonization yield, and activation degree all affect pore volume, pore-size distribution, and compaction density. Biomass-derived routes are attractive in terms of cost and sustainability, but ash content, impurity composition, and feedstock fluctuation may weaken structural reproducibility. For Si/C anodes, CVD-based silicon incorporation is effective for obtaining uniform nanosilicon distribution, yet the cost of silicon-containing gases, safety control, exhaust-gas treatment, and deposition uniformity still increase scale-up difficulty. For sodium-ion battery hard-carbon routes, low-cost precursors become practically competitive only when ash control, carbon-layer consistency, compaction density, and initial Coulombic efficiency are simultaneously satisfied. Therefore, future industrial evaluation should shift from whether carbon aerogel-derived anodes can be prepared to whether they can be produced with stable quality, low cost, and reduced environmental burden, while further emphasizing compaction density, areal capacity, volumetric capacity, long-term impedance evolution, and full-cell validation under high-loading electrode conditions.

## 6. Conclusions and Perspectives

This review systematically summarizes the controllable synthesis, heteroatom doping, and alkali-metal-ion battery applications of carbon aerogels. Sol–gelation, drying, carbonization, and activation determine pore-size distribution, pore connectivity, framework shrinkage, surface area, and carbon ordering. Heteroatom doping further regulates charge distribution, defect chemistry, active sites, and interfacial reactions. On this basis, the functions of carbon aerogels in lithium-, sodium-, and potassium-ion batteries are compared from the perspectives of ion transport, electron conduction, volume-stress accommodation, interface stabilization, and storage mechanism matching. These discussions indicate that carbon aerogels should not be designed simply as high-surface-area porous carbons. Their electrochemical performance depends on the coordinated regulation of accessible pore channels, stable doped sites, continuous conductive networks, and electrode/electrolyte interfaces.

Future research should pay more attention to mechanism-guided structural design under practical electrode conditions. Gradient heteroatom doping may provide a useful route for spatially regulating surface polarity, electronic conductivity, and interfacial reactivity within the same aerogel framework. Defect engineering should also move from increasing total defect density to controlling defect type, location, and stability, because excessive defects can promote irreversible ion trapping, electrolyte decomposition, and unstable SEI growth. For thick electrodes and high-areal-capacity designs, carbon aerogels need to maintain open ion pathways, mechanical integrity, and electronic percolation after electrode densification. Binder-free or self-supporting aerogel architectures are also worth further exploration because they may reduce inactive components and improve interfacial contact, but their mechanical robustness, manufacturing reproducibility, and compatibility with roll-to-roll processing still require systematic verification. From an application perspective, the main gaps are no longer limited to laboratory capacity improvement. Long-term impedance evolution, SEI stability, electrode swelling, mechanical fatigue, thermal safety, gas evolution, and failure behavior under lean-electrolyte or full-cell conditions remain insufficiently understood. Resin-based precursors offer good structural controllability, but their cost and environmental burden need to be reduced. Biomass-derived precursors are attractive for low-cost production, yet ash control, compositional fluctuation, carbonization yield, and pore-structure consistency remain major barriers. Therefore, future evaluation should include compaction density, initial Coulombic efficiency, areal capacity, volumetric energy density, high mass loading, limited electrolyte, full-cell or pouch-cell validation, and processing compatibility. Carbon aerogels are more likely to move from laboratory materials to practical energy storage electrodes only when pore architecture, doped-site stability, mechanical durability, interfacial safety, production cost, and scalable manufacturing are addressed together.

## Figures and Tables

**Table 1:** Comparison of representative reviews on carbon aerogels.

Authors (Year)	Main Scope	Highlights/Limitations	Ref.
Tomić et al. (2025)	Polymerization; drying; carbonization; precursors; doped and metal-decorated carbon gels; H_2_O_2_ production; supercapacitors	Broad sustainable energy/environment scope; limited discussion of alkali-metal-ion battery anodes and ion-storage mechanisms	[22]
Bari and Jeong (2024)	Hierarchical pores; surface polarity; N/O/S/P/B heteroatom doping; carbon microstructure; synthesis strategy; energy-related applications	General energy-application perspective; insufficient ion-specific storage analysis and practical battery evaluation	[23]
Gao et al. (2025)	Carbon-based aerogel preparation; graphene aerogels; separators; interlayers	Li–S chemistry-centered scope; limited relevance to alkali-metal-ion battery anode design	[24]
Li et al. (2025)	Carbon aerogel-based electrochemical systems; charge-storage mechanisms; synthesis methods; heteroatom doping; composite engineering; supercapacitors; battery hybrids; electrocatalysis	Broad multifunctional scope; dispersed discussion of alkali-metal-ion battery anodes and practical electrode evaluation	[25]
Li et al. (2024)	Biomass precursors; cellulose; lignin; chitosan; biomass waste; supercapacitors; hybrid capacitors; metal-ion batteries; fuel cells	Biomass-derived material focus; limited coverage of non-biomass carbon aerogels and general anode design principles	[26]
Li et al. (2026)	Fabrication methods; structural design; conductivity enhancement; chemical modification; surface/interface tailoring; supercapacitors; batteries; electrocatalysts	Preparation-oriented focus; limited analysis of ion-dependent anode mechanisms and scalability-oriented electrode design	[27]
This review	Controlled synthesis; pore regulation; heteroatom doping; Li/Na/K-ion battery anodes; practical electrode evaluation; industrial scalability	Mechanism-oriented framework; Li/Na/K-specific design principles; structure–doping–transport–interface linkage; electrochemical performance comparison; scalability-oriented perspective	

**Table 2:** Effects of different pore-regulation methods on pore-size distribution, specific surface area, and electrochemical properties of carbon aerogels.

Method	Effect on Pore-Size Distribution	Effect on Specific Surface Area	Effect on Electrochemical Properties
Sol–gelation	Initial pore-network formation; particle-size control; micro/mesopore regulation; pore-connectivity construction	Accessible surface formation; pore-accessibility control; improved structural uniformity	Improved electrolyte infiltration; faster ion diffusion; enhanced charge transport; optimized rate performance
Drying	Wet-gel pore retention; shrinkage and collapse suppression; oriented pore and macropore formation	Pore-volume retention; surface-area preservation; area loss induced by drying stress	Higher interface utilization; optimized ion-transport pathways; improved mass transport in thick electrodes; enhanced cycling stability
Carbonization	Micropore development; framework shrinkage; pore-coalescence risk; carbon-structure fixation	Surface-area increase at moderate temperature; accessible-area loss after over-carbonization	Higher conductivity; lower charge-transfer resistance; regulated storage sites; weakened interface utilization at excessive temperature
Activation	Closed-pore opening; micro/mesopore reconstruction; improved pore connectivity; over-etching risk	Large surface-area increase; higher micropore fraction; surface-area loss after overactivation	Increased capacity/capacitance; stronger interfacial storage; regulated ion diffusion; improved or restricted rate performance

**Table 3:** Synthesis parameters and pore-structure characteristics of carbon aerogels reported in representative studies.

Precursor	Catalyst	Precursor-to-Catalyst Ratio	Drying Method	Carbonization Temperature/°C	Activation Method	Main Pore Size/nm	Specific Surface Area/m^2^ g^−1^	Pore Volume/cm^3^ g^−1^	Ref.
Resorcinol/ formaldehyde	Na_2_CO_3_; HNO_3_	R/C = 500	Ambient-pressure drying; supercritical drying	950	No activation	ACCA: 1.71; BCCA: 1.90	ACCA: 461.59; BCCA: 395.90	ACCA: 0.266; BCCA: 0.225	[31]
Resorcinol/ formaldehyde; GCNs	GCNs; Na_2_CO_3_	R/GCNs = 3:1; R/C = 500	Ambient-pressure drying	900	No activation for the main sample; some samples underwent CO_2_ physical activation + NH_3_ activation	15	539	0.786	[33]
Resorcinol/ formaldehyde; chitosan	Acetic acid; Na_2_CO_3_	R/acetic acid = 2.5; R/C = 500	Supercritical drying	1000	No activation	9.5	636	1.51	[34]
Resorcinol/ formaldehyde	Na_2_CO_3_	R/C = 1000	Supercritical drying	700	KOH chemical activation	1.78	684	0.095	[38]
Resorcinol/ formaldehyde	No gel catalyst	Not reported	Supercritical drying	700	No activation	3.13	698.2	10.86	[39]
Kraft lignin/ cellulose nanofibers; graphene dots	No gel catalyst	Not reported	Freeze-drying	1000	No activation	1.77	503	0.222	[41]
Resorcinol/ formaldehyde; cotton fibers	Na_2_CO_3_	R/C = 200	Ambient-pressure drying	1000	No activation	3.9	730	0.89	[42]
Resorcinol/ formaldehyde; tetraethyl orthosilicate	Na_2_CO_3_; HCl	R/C = 500	Ambient-pressure drying	1500	No activation	18	299.3	0.77	[43]
Phenolic resin; polyurethane-urea oligomer	NaOH; H_2_SO_4_	Not reported	Ambient-pressure drying	1400	No activation	1.24	Not reported	Not reported	[45]
Resorcinol/ formaldehyde	HCl	R/HCl ≈ 13	Supercritical drying	800	CO_2_ physical activation + KOH chemical activation	0.97	3010	1.39	[47]
Alginate/pullulan	No gel catalyst	Not reported	Freeze-drying	900	CaCO_3_ in situ activation–graphitization + HCl acid washing	1	327.4	0.28	[50]

**Table 4:** Mechanisms, advantages, and limitations of single heteroatom-doped carbon aerogels.

Doping Element	Main Regulation Mechanism	Effects on Charge Transport, Interfacial Chemistry, and Ion Adsorption	Advantages	Limitations and Design Concerns	Ref.
N	N configuration regulation; charge redistribution; polarity modulation; pseudocapacitive site construction	Enhanced electron transport, improved electrolyte wettability, stronger defect/edge ion adsorption, interfacial redox contribution	Better wettability; more active sites; faster charge transfer	Temperature-sensitive sites; N loss at high temperature; dependence on pore accessibility	[55]
P	P–C/P–O bonding; defect generation; interface regulation; ion-diffusion modulation	Enlarged interlayer spacing, reduced ion-diffusion resistance, improved charge transport, enhanced surface polarity, regulated SEI formation	Improved charge transfer; faster ion diffusion; better rate and cycling performance	Uncontrolled P distribution; pore blockage risk; reduced interface utilization	[57]
B	B–O/B–C bonding; charge redistribution; polarity regulation	Electron-deficient active centers, localized charge polarization, enhanced ion adsorption, improved interfacial wettability, increased diffusion resistance	Higher thermal stability; better wettability; enhanced ion adsorption	Limited doping content; higher diffusion resistance; unclear active-site contribution	[59]
S	C–S bonding; polar site formation; active species anchoring	Enlarged local interlayer spacing, polar C–S/C–SOx sites, enhanced ion affinity, stronger interfacial anchoring, modified charge-transfer kinetics	Stable interfaces; suppressed species migration; improved reaction reversibility	Poor thermal stability; difficult site control; distinction from sulfur loading required	[61]

**Table 5:** Structural requirements and design priorities of carbon aerogels in different electrochemical energy storage systems.

Energy Storage System	Main Storage Mechanism	Main Limitations	Roles of Carbon Aerogels	Design Priorities	Ref.
**Lithium-ion batteries**	Li^+^ insertion/extraction; alloying reactions; conversion reactions	Large volume variation of Si, SnO_2_, sulfides, and selenides; repeated SEI rupture; loss of electronic contact; low initial Coulombic efficiency	Percolated conductive skeletons; stress-buffering matrices; active-phase dispersion and anchoring; volume expansion accommodation; interface stabilization; fast Li^+^ transport	Continuous electron pathways; sufficient void space; stable interfacial coating; hierarchical pores; suppressed parasitic reactions; robust active-phase loading	[73]
**Sodium-ion batteries**	Na^+^ insertion/adsorption; surface pseudocapacitive storage; conversion reactions	Large Na^+^ radius; sluggish diffusion kinetics; difficult carbon interlayer insertion; stress accumulation in active phases; unstable SEI; low initial Coulombic efficiency	Open ion transport channels; shortened Na^+^ diffusion pathways; interlayer-spacing regulation; active-phase buffering; enhanced surface capacitance; improved interfacial kinetics	Enlarged interlayer spacing; interconnected mesopores/macropores; moderate defect density; high framework conductivity; strong carbon skeleton; controlled rather than excessive surface area	[79]
**Potassium-ion batteries**	K^+^ insertion/extraction; surface adsorption; conversion reactions	Larger K^+^ radius; severe insertion-induced strain; high migration barrier; unstable SEI; irreversible reactions from excessive defects	Stable interlayer environment; collapse-resistant aerogel framework; stress accommodation space; stable K^+^ adsorption sites; charge transport pathways; SEI regulation through doping/electrolyte coupling	Stable expanded interlayer spacing; oriented pore channels; stable dopant sites; strong framework integrity; moderate defect density; balanced conductivity, wettability, and mechanical compliance	[84]

**Table 6:** Representative electrochemical performance of carbon aerogel-based anodes in alkali-metal-ion batteries.

Battery System	Specific Capacity	Half-Cell Cycling Performance	Full-Cell Cycling Performance	ICE	Ref.
LIB/SIB	LIB: initial discharge/charge capacities of 1618.4/1346.7 mAh g^−1^; SIB: 534.4 mAh g^−1^ at 0.1 A g^−1^	LIB: 1318 mAh g^−1^ after 50 cycles at 0.1 A g^−1^; SIB: 534.4 mAh g^−1^ after 100 cycles at 0.1 A g^−1^	Not reported	LIB: 83.2%; SIB: 80%	[74]
LIB	541 mAh g^−1^ after 1000 cycles at 1 A g^−1^; 505 mAh g^−1^ at 2 A g^−1^; initial discharge/charge capacities of 994/840 mAh g^−1^	541 mAh g^−1^ after 1000 cycles at 1 A g^−1^; 505 mAh g^−1^ after 200 cycles at 2 A g^−1^	Not reported	84.5%	[75]
LIB/SIB	LIB: initial capacity of 941.1 mAh g^−1^; SIB: initial discharge capacity of 750.2 mAh g^−1^	LIB: 768.5 mAh g^−1^ after 700 cycles at 1 A g^−1^; SIB: 454.9 mAh g^−1^ after 300 cycles at 1 A g^−1^ and 438.8 mAh g^−1^ after 500 cycles	123.2 mAh g^−1^ after 100 cycles; 286.6 mAh g^−1^ after 60 cycles	LIB: 67.0%; SIB: 76.0%	[76]
LIB	Initial discharge/charge capacities of 531/424.9 mAh g^−1^; 277 mAh g^−1^ at 2 C	474.7 mAh g^−1^ after 200 cycles at 0.5 C	Not reported	80.02%	[77]
SIB	700 mAh g^−1^ at 50 mA g^−1^ after 100 cycles; 200 mAh g^−1^ at 5 A g^−1^	700 mAh g^−1^ after 100 cycles	Not reported	89.18%	[80]
SIB/PIB	SIB: initial discharge/charge capacities of 1080/530 mAh g^−1^; PIB: initial discharge/charge capacities of 1400/420 mAh g^−1^	SIB: 673 mAh g^−1^ after 80 cycles at 0.1 A g^−1^; PIB: 349 mAh g^−1^ after 80 cycles at 0.1 A g^−1^	Not reported	SIB: 48.5%; PIB: 30%	[81]
SIB	282 mAh g^−1^ at 0.1 A g^−1^; 175–178 mAh g^−1^ at 5 A g^−1^	238 mAh g^−1^ after 7000 cycles at 0.5 A g^−1^	Not reported	24.3%	[82]
PIB	Initial discharge/charge capacities of 376.8/174.1 mAh g^−1^	105 mAh g^−1^ after 300 cycles at 0.02 A g^−1^	Not reported	46.2%	[85]
PIB	507 mAh g^−1^ after 100 cycles and 106 mAh g^−1^ after 3000 cycles at 5 A g^−1^	106 mAh g^−1^ after 3000 cycles at 5 A g^−1^	Not reported	Not reported	[86]
SIB/PIB	SIB: 298 mAh g^−1^ at 0.1 C; PIB: 258 mAh g^−1^ at 0.1 C	SIB: 220 mAh g^−1^ after 200 cycles at 0.5 C; PIB: 82.7% capacity retention	SIB: 71.3% after 100 cycles and 206 mAh g^−1^ at 2 C; PIB: 194 mAh g^−1^ at 0.1 C, 84 mAh g^−1^ at 1 C, and 100 cycles at 0.5 C	SIB: 44%; PIB: 34%	[87]
PIB	227.4 mAh g^−1^ at 100 mA g^−1^ after 100 cycles; 134 mAh g^−1^ at 1000 mA g^−1^	125 mAh g^−1^ after 1000 cycles at 500 mA g^−1^	Not reported	36.3%	[88]
PIB	Initial discharge/charge capacities of 1672.4/441.6 mAh g^−1^; reversible charge capacity of 420 mAh g^−1^ at 0.05 A g^−1^	274.8 mAh g^−1^ after 200 cycles at 0.05 A g^−1^	Not reported	26.4%	[89]

## Data Availability

No new data were created or analyzed in this study.
